# Investigation of hydrochemical characteristic, water quality and associated health risks of metals and metalloids in water resources in the vicinity of Akamkpa quarry district, southeastern, Nigeria

**DOI:** 10.1186/s12932-024-00090-y

**Published:** 2024-09-12

**Authors:** George E. Ikpi, Therese N. Nganje, Aniekan Edet, Christopher I. Adamu, Godswill A. Eyong

**Affiliations:** 1 Department of Geology, University of Calabar, Calabar, Nigeria; 2 Department of Geoscience, University of Uyo, Uyo, Nigeria

**Keywords:** Geochemical characteristics, Water resources, Water quality, Human health risk, Quarry district, Southeastern Nigeria

## Abstract

Quarrying of rock aggregates generates produced water that, if not handled properly will be a source of pollution for nearby water bodies, thus affecting the chemistry of the water. This study examined the chemistry, impact of quarrying activities on water resources and the health consequences/risks posed by ingestion of the water by humans in the Akamkpa quarry region in southeastern Nigeria. Thirty (30) water samples consisting of pond water, stream water, hand dug wells, and borehole samples were collected and analyzed for their physicochemical parameters using standard methods. The results obtained from the analyses indicated that the water was moderately acidic, fresh, and not salty, with many parameters below the recommended standards with Ca^2+^, and HCO_3_^−^ being the dominant ions present in the water resources. Rock weathering processes including silicate weathering as illustrated by hydrochemical facies, cross plots, and Gibbs diagrams are the dominant mechanisms influencing the quality and major ions chemistry of the water resources with minor contributions from dissolution, anthropogenic activities, and ion exchange. Ca-Mg-SO_4_-Cl and Na–K-HCO_3_^−^ are the most important water types. Although the water quality index shows that the water is suitable for human use and irrigation, the mean values of As, Cd, Pb, and Se are above the acceptable limits. Additionally, the calculated contamination factor revealed the water resources are moderate to highly contaminated by As, Cd, Cr, Mo, Pb, Sb, and Se, and are therefore unsuitable for consumption with regards to these parameters. However, the residual sodium carbonate and water hazard index (WHI) values showed that 38% to 90% of sites in the quarry area were unsuitable for cultivation, 10–30% were in the low to medium impact category, and 60% were classified as risky and are from high to very high impact category. A non-cancer study of inhabitants living in the vicinity of the quarry area indicated that 6.7% of the sites have values greater than one, indicating that it may endanger the health of the people. Therefore, constant monitoring of the water quality is recommended as long-term use of contaminated water can harm humans, plants, and soils.

**Supplementary Information:**

The online version contains supplementary material available at 10.1186/s12932-024-00090-y.

## Introduction

**Fig. 1:**
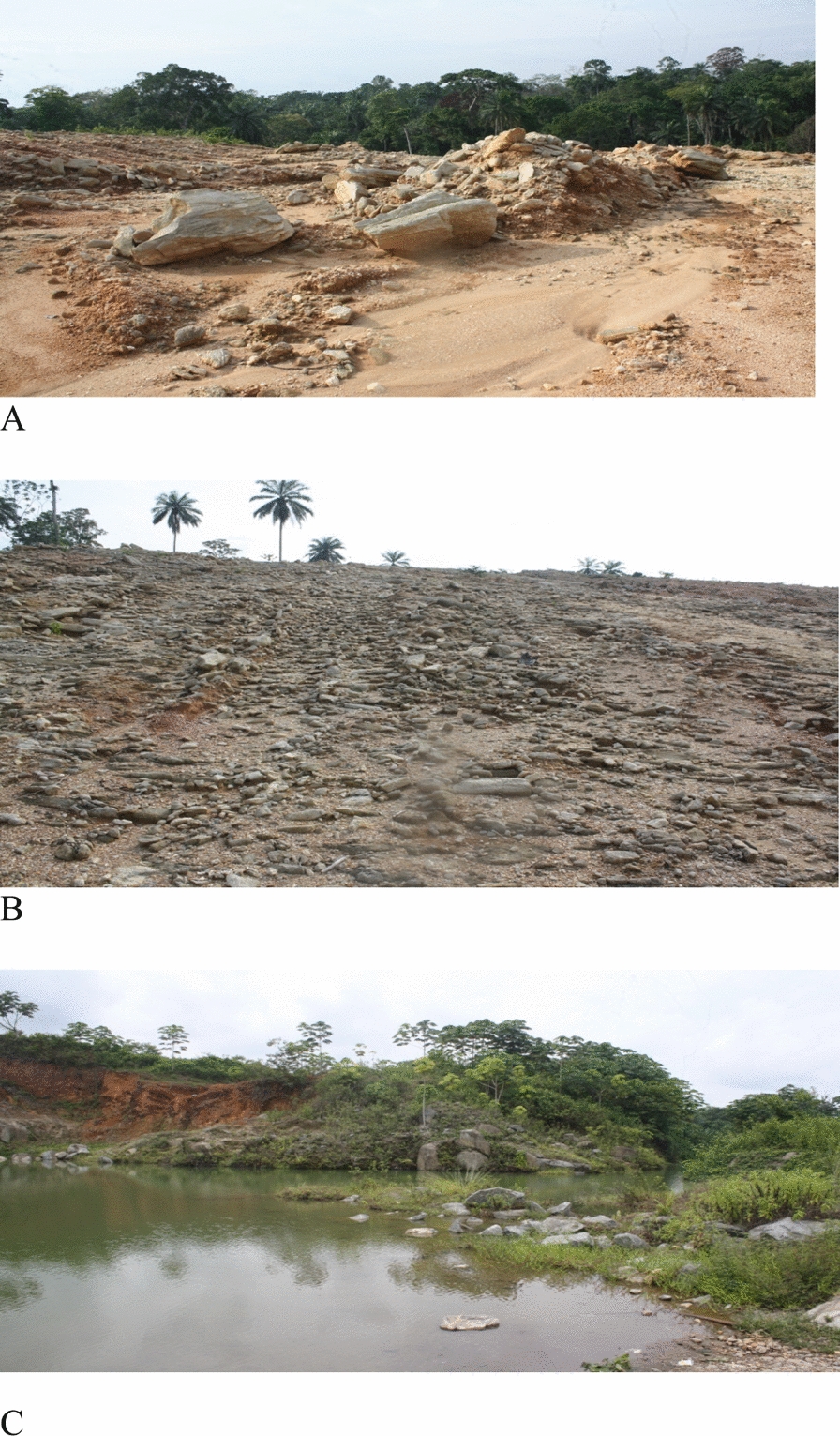
**a**, **b** Scattered quarry waste materials and **c** Flooded quarry pit

Water is an important resource for the health of urban ecosystems for residential, construction, and industrial uses, and pollutants from different and unrelated sources have the potential to harm physical, chemical, and aquatic life [30]. In addition to natural sources of pollution, anthropogenic activities such as rock quarrying also pose challenges to sustainable water resources*.* The various activities of the quarry include clearing/removal of vegetation and overburden materials, excavation, blasting, transportation, and crushing of rocks [54] producing waste materials (Fig. 1a). These quarry materials include highly visible, dispersed fragments of materials such as granite, gneiss, diorite, limestone, and shale in piles and heaps around the quarry sites have become a source of contamination in the environment. Often these quarries do not have preparedness plans to combat environmental pollution or properly manage quarry waste materials, and sometimes quarries are abandoned without proper management (Fig. 1b). Some of these waste materials contain pyrite and other related minerals that can increase the acidity of water if they encountered it. The ponds (Fig. 1c) created by quarrying activities, during heavy rainfall, and high tides flood huge volumes of water which can cause the release of pollutants (Pb, Cd, Cr, Cu, Zn, Mn, Fe) into the surrounding water. Some metals such as Cu, Fe, Mn, and Ni are essential micronutrients for plants and microbes, while others such as As, Pb, Cd, are harmful at high concentrations [65]. Humans are exposed to high levels of metal(loid)s through contaminated water, the use of contaminated water to prepare food, and the irrigation of food crops. In addition, mining, and quarrying expose rock surfaces, and are prone to the weathering of contaminants into nearby water surface bodies leading to pollution and harm to water, including human health, after long periods of water consumption. It is known that heavy metals pose a threat to human health due to their toxicity, persistence, bioaccumulation in foods, and non-degradable nature in the environment [12, 51]. It is a fact that both surface and groundwater sources are dependent on each other. Many surface streams receive a major portion of their flow from groundwater. On the other hand, water coming from surface waterways is the most important source feeding groundwater. Therefore, the two sources of supply are interrelated and the use of one may affect the availability of the other [61]. Because water is scarce, irreplaceable, and essential for global health, water in the study area serves as a source of drinking water and other domestic purposes for quarry workers and neighboring communities who are subsequently exposed to health risks arising from problems because of using these contaminated water bodies.

**Fig. 2:**
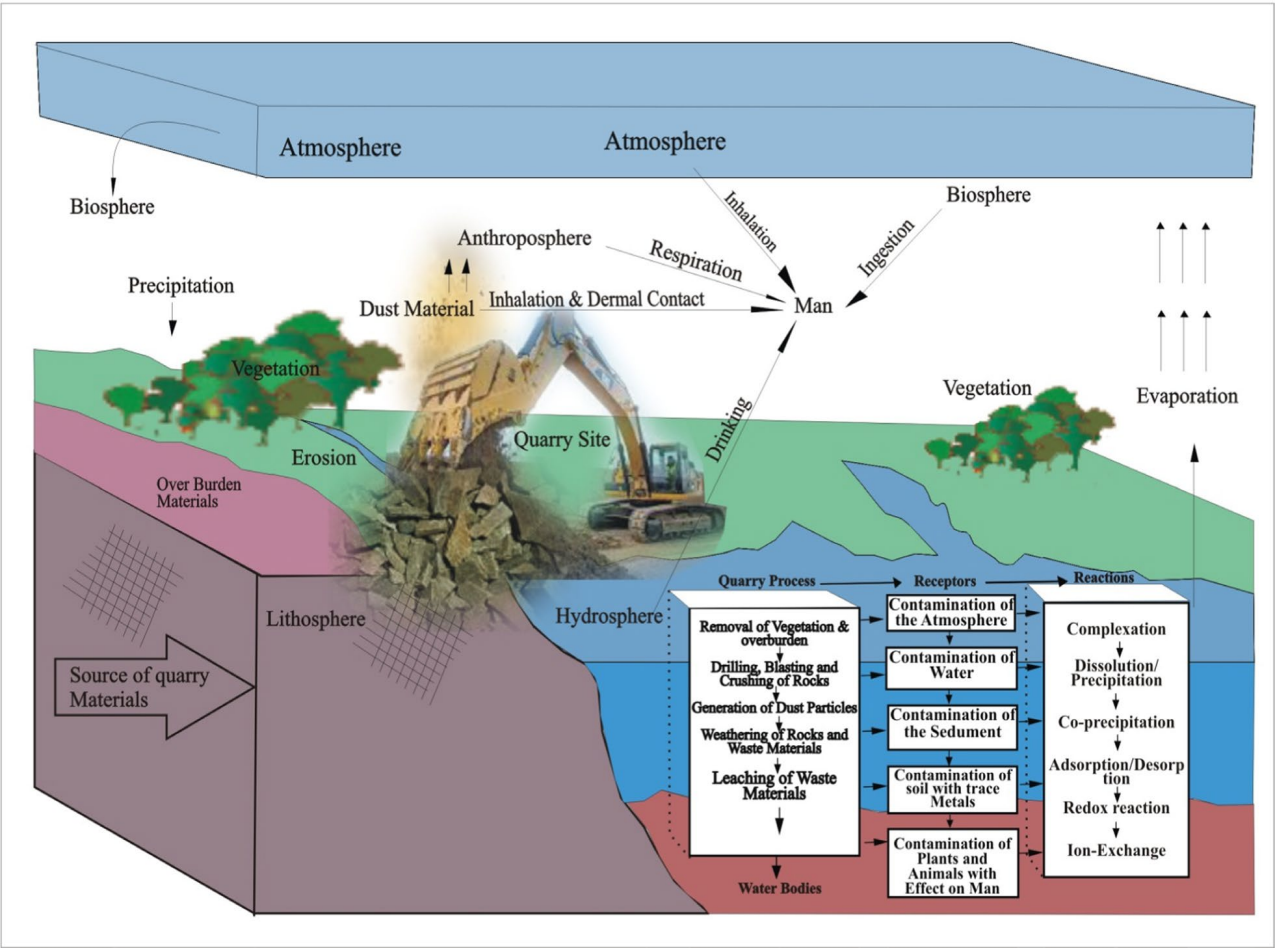
Conceptual model showing quarrying activities, processes and reactions (Modified from Geochemical Modeling- A Review of Current Capabilities and Future [15]

Studies in some parts of the globe have shown that indiscriminate disposal of quarry wastes are common sources of metal(loid)s pollution in water resources [46, 55] and has the potential to affect human health through drinking water pathway. Impact of pollution from marble, sandstone, and limestone quarries of water sources by chromium and zinc in western Nigeria have been reported by Afeni et al. [4] and [9]. All these studies indicate the negative impact of quarry activities on environmental resources. Available data in the study area was limited to quality assessment of water in different seasons, spatial variation, and changes [24] while the work of [2, 3] study focused on the impact of abandoned barite mines on water, Land, and sediment. Most of the studies conducted in part of the basement area by Ekwere and Edet [27], Sikakwe and Ilaumo [68, 69] are based on the heavy metal evaluation index (HEI), and heavy metal pollution index (HPI). However, none of these studies was focused on water hazard impact and associated risk to human health in the basement and sedimentary sector of the study area where limestone is currently mined for cement production. Before quarried activities in the study area, local people were engaged in agricultural activities, including crop production, fishing, and other important sources of income. However, recently intensive agriculture in the region has been replaced by quarry activities. Indiscriminate disposal of quarry materials, including ash dumping, has negatively affected environmental quality. The relationship between water sampling, the environment, quarry operations, and other processes and humans can be affected by the movement and leaching of pollutants into surface and groundwater (Fig. 2). Runoff from quarry dumps and quarry ponds are sometimes used for cooking, drinking, bathing, and fishing by people living in the vicinity of the quarry sites. Thus, it is possible for humans and animals that depend on these water sources to accumulate these pollutants from contaminated water, ultimately posing a risk to users. However, evaluating the composition of water resources is important in terms of assessing their suitability for use, domestic use, irrigation, agricultural purposes, and the effects of consumption on human health. In this study, in addition to irrigation purposes, the geochemistry, toxicity and human health evaluation of metals and metalloids found in various water resources in the study area were also investigated. Therefore, this study is the first covering quarries in basement, and sedimentary terrains, although most of the samples were from the basement terrain*.* Consequently, it has become necessary to assess the environmental impact, quart siting, and quarry operations on the quality of various water bodies around quarries in parts of the south-south regions of Nigeria though.

Therefore, the main purpose of this study is as follows: document the geochemical characteristics of the water resources in the Akampka quarry area, examine the quality of water, evaluate the impact and health risk of metals and metalloids, and suitability for drinking, domestic use, irrigation use, and agriculture. The results from this study will further contribute to the quality, monitoring, and management of various water resources in the study area.

### Study area description

**Fig. 3:**
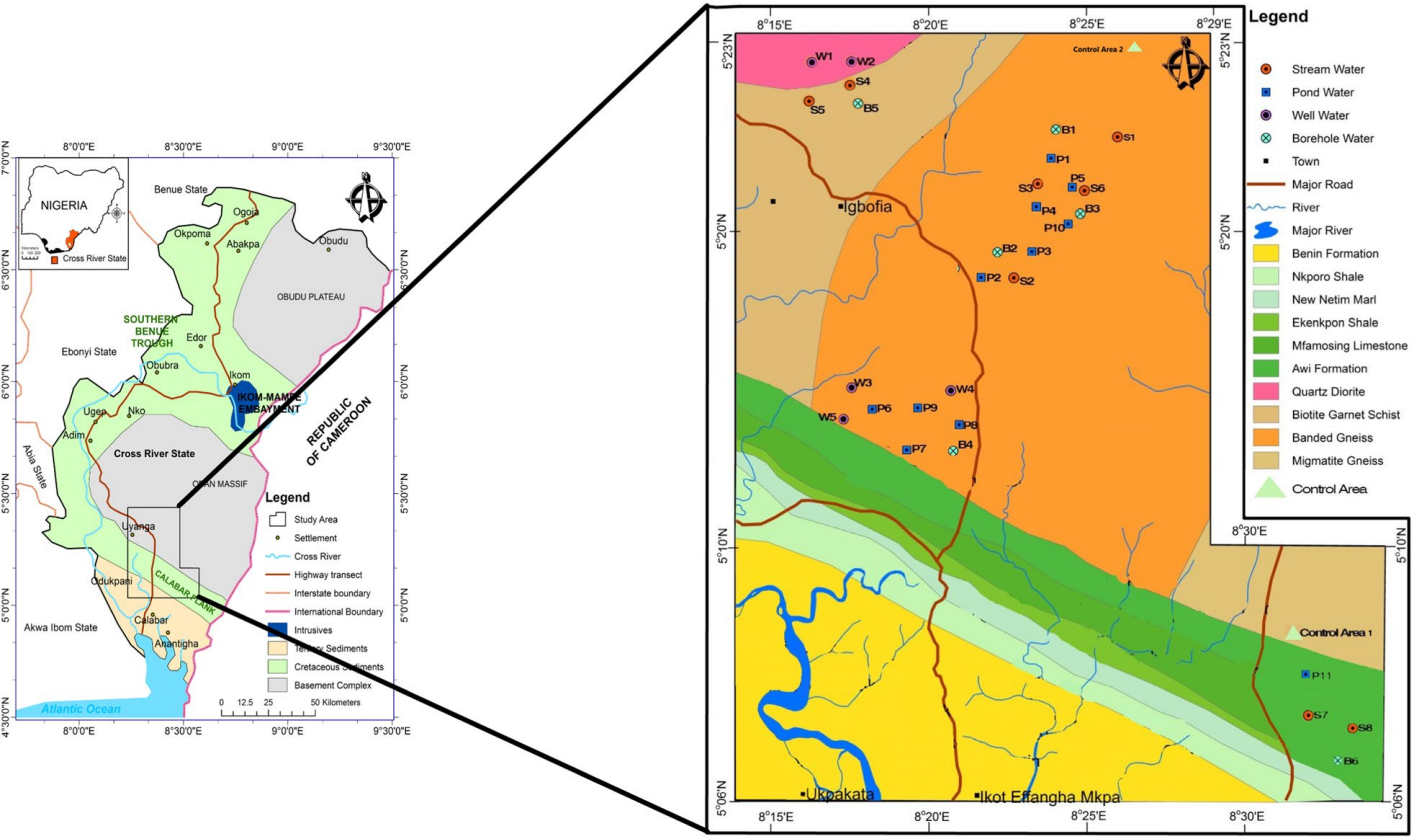
Study area showing geology and sample location. Modified from Nigerian Geological Survey Agency, 2020)

Akampka quarry area is located between Latitudes 05° 06’ and 05° 23’ North and Longitudes 08° 15’ and 08° 30’ East. It includes parts of the basement area of the Oban massif and the sedimentary terrain of the Calabar flank in southeastern Nigeria (Fig. 3). It is located in the Nigerian climatic zone, where annual rainfall varies between 180 and 200 cm and annual temperature is between 25 and 30 °C [39]. There are two main seasons in the region, the wet season (April–October) and the dry season (November-March) with a short break in August (August break). Humidity in this region is always high; it is usually over 90% in the morning, over 100% at night during the rainy season, and drops to 70% during the dry season. The surface of the area is generally curved and undulating and consists of alternating valleys. The geographical structure of the region runs in an indefinite direction and separates the low-lying areas from moderate relief landmarks. The region is heavily irrigated by the Cross River and its tributaries such as Ayipojong-Ita, and Etap-Ayip, and some long-standing rivers such as Ikpaya, Iwiri, Monayip-Netim, and others. Rivers and streams often move quickly in their headwaters with little or no bedload. Typically, the flow pattern in this region is dendritic and, in some cases, linear, indicating structural control. Most rivers in the region are seasonal: they flow heavily during the wet season and usually dry up during the dry season [1].

Geologically as shown on Table 1, the basement area is made up of mainly gneisses, schists, amphibolites pegmatites, granites, and granodiorites, of varied composition. The main lithologic unit of the sedimentary terrain known as the Calabar Flank region of the study area includes sandstone, limestone, shale, and marl. The Calabar Flank holds very low prospects for groundwater because of the presence of shale that constitute thick aquitards, but where the shale is extensively fractured, it can form good groundwater reservoirs. Nevertheless, one of the potential sources of aquifer recharge in the area is surface precipitation. However, the conglomerates, sandstones, and limestone constitute water-bearing units in the area. In the basement area, the ground water occurrence is through fractures, joints fissures [26]. The water-bearing units consists of an upper highly weathered layer, a middle slightly-moderately weathered layer, and a lower fractured bedrock with the water table being highly variable and ranging between < 1.0 m in the northern part and 10.0 m in the southern part of the massif with yield in the range of 10–200 m^3^ d^−1^[53].

**Table 1:** Location and physical setting of the quarry sites

S/N	Name of quarry	Lat.(N)	Long.(E)	Elevation (m)	Year of establishment	Formation	Lithology	Age	Area (hectare)	Production /day(Tones)	Status during Sampling	Current Status	Year abandoned	Duration of quarry
1	Unicem	05 94.64	08 31.951	35	2002	Mfamosing	Marl, Shale Limestone	Albian	50	6,250	Active	Active till date	–	22
2	Crush Rock	05 21.83	08 21.623	126	1976	Basement	Gneiss, Schist	Precambrian	35	2000	Active	Abandoned	2022	46
3	Gitto	05 22.30	08 21.943	118	2005	Basement	Gneiss, Granodiorite	Precambrian	25	400	Active	Abandoned	2022	17
4	Cewopi	05 23.06	08 21.038	101	2014	Basement	Gneiss, pegmatite	Precambrian	22	200	Active	Abandoned	2022	8
5	Crush & Pave	05 18.28	08 20.797	101	2009	Basement	Schist, Granite	Precambrian	25	800	Active	Abandoned	2023	14
6	RCC	05 18.33	08 20.484	117	2007	Basement	Schist, Granite	Precambrian	25	800	Active	Abandoned	2022	15
7	Expanded	05 18.23	08 22.027	97	2009	Basement	Schist, Quartzite	Precambrian	25	1000	Active	Abandoned	2022	13
8	S & V	05 18.75	08 24.980	115	2008	Basement	Schist, Gneiss	Precambrian	22.5	600	Active	Abandoned	2023	15
9	Enerco	05 15.35	08 18.814	115	2008	Basement	Gneiss, pegmatite	Precambrian	15.5	400	Abandoned	Abandoned	2018	10
10	Cottab	05 15.16	08 20.414	130	2012	Basement	Granodiorite, pegmatite	Precambrian	18.6	500	Abandoned	Abandoned	2016	4
11	Japaul	05 14.84	08 19.515	114	2011	Basement	Granite, Gneiss	Precambrian	25.8	600	Active	Abandoned	2021	10
12	Sermatech	05 21.15	08 16.525	111	2012	Basement	Granodiorite Granite	Precambrian	16.8	300	Abandoned	Abandoned	2019	7

### Quarry activities

The current study considered twelve quarry sites developed between 1976 and 2014 covering an average area of 25.5 hectares with an average production of 1154 tonnes/day. Geologically, eleven (11) of these quarries were in the basement terrain while only one (1) was within the sedimentary terrain within the Calabar Flank (Fig. 3) of the study area some of which are active and abandoned. A summary description of these quarries including the duration of the quarrying activities as well as the status and their production capacities are also outlined in Table 1. Quarrying activities have increased over the years within the quarrying district of Akamkpa. Currently, there exist more than 30 quarries in the study area. The high number of quarries is in response to the increasing demand for crushed rocks by construction industries due to the upsurge in infrastructural development in Nigeria.

A total of thirty (30) samples and two (2) control samples were collected from different locations during the rainy season. The water samples were collected in 250 ml (anions analyses) and 50 ml (cations analyses) clean plastic bottles that were thoroughly washed and rinsed with deionized water in the laboratory and then in the field to prevent contamination. Water samples were collected from quarry ponds (P), nearby streams (S), hand-dug wells (W), borewells (B) surface water control (SW) and groundwater control (GW), Fig. 3. In ponds, water bottles were placed under water to collect samples that did not contain films that could be a source of heavy metals [36]. Two samples were collected at each site and labeled to avoid mixing. One part of the sample was used to determine physical parameters and anions, and the second part was used to determine cations. These samples were passed through 0.45 μm filter paper to remove suspended solids that could dissolve and affect metal concentrations. Samples for cations analyses were acidified with 2 mL of HNO_3_^−^ acid to keep metal ions in solution. Water samples for anion analysis were stored in a refrigerator at 4 °C for a week before transportation to the Laboratory for analysis to reduce the concentration of dissolved compounds. Physical parameters of water such as temperature, conductivity (EC), and pH were measured in the field using a thermometer and pH meter. Laboratory Analysis of Cations was carried out by Inductively Coupled Plasma Mass Spectrometry (ICP-MS), Perkin Elmer Sciex at the National Agency for Food and Drug Administration (NAFDAC), Lagos, Nigeria. Total hardness (TH) and anions were analyzed by titrimetric and chromatographic methods at the Institute of Oceanography (IOC), University of Calabar, Nigeria. A quality control measure was also used. Each analysis was performed in triplicate and the mean value was recorded. The control scheme used, involved two samples and in-house reference materials. Calibration standards for the Spectrophotometer was done using prepared serial solution from traceable stock and verified against in-house reference materials, and the resulted concentrations of the anions, metals, and metalloids were reported in mg/L and µg/L.

## Data handling

### Statistical analyses

Descriptive statistics were performed utilizing the statistical package STAISTICA [60]), and Excel spreadsheet. Mineral phases were computed using the computer program PHREEQC. It aimed to evaluate the role of mineral dissolution to reveal the potential of mineral controls on water chemistry by calculating the distribution of aqueous species and mineral saturation indices [25]). Saturation indices (SI) indicate whether a water sample is saturated or unsaturated for a particular mineral.

1 $${\text{SI}}\, = \,{\text{log}}_{{{1}0}} \left( {{\text{IAP}}/{\text{K}}_{{{\text{SP}}}} } \right)$$

where Ksp = solubility product of at a given temperature.

IAP = Ionic Activity Product.

SI predicts the mineral water balance and water–rock interaction [86]. If the SI is zero, the water is saturated with certain minerals. An SI of less than zero indicates an inadequate saturation or undersaturation(dissolution) for a particular mineral and may indicate the presence of water from a rock that does not contain enough mineral to rapidly dissolve or penetrate/infiltrate and such minerals will continue to change with groundwater [86]. SI above zero indicates an oversaturated (precipitated) state for the mineral phase [64] and does not dissolve most minerals. Oversaturated water refers to groundwater resources that have sufficient mineral salts and sufficient residence time to reach equilibrium [7]. However, significant changes in alkalinity and SI values are related to time. Therefore, the SI of water is close to zero (−0. 5 and + 0. 5) will be nearly neutral, so water will not tend to dissolve or precipitate the mineral [34], which represents the equilibrium state of the mineral [86].

Principal component analysis (PCA) was used to obtain quantitative data and group the measured factors for interpretation [5]. The aim of this was to determine the source of the ions, metals, and metalloids in the water and to evaluate the correlation coefficient between them. The varimax regression method was used to determine the relationship between the data and the most important factors [52]. The data are obtained by transforming the original data set into the standard version due to variance, creating a new set of uncorrelated pseudo variables known as principal components (PC). PCs were identified by running screen plots with eigenvalues  > 1 (Kaiser standard method) [48].

### Hydrogeochemical evaluation

Hydrogeochemical facies of water samples were evaluated using the Piper diagram [59], Using the Piper diagram, the origin, structure, and chemical interactions between cations and anions dissolved in the waters are analyzed. Hydrochemical composition is mainly affected by lithology, residence time, and regional groundwater flow pattern [21]. Water can be classified as bicarbonate, sulfate, and chloride according to its chemical composition [11]. Gibbs diagrams [33] and cross-plots have also been used to determine the type and processes controlling water chemistry. Also, PCA, was used to determine the nature of hydrochemical interactions between water and the environment [10].

### Water quality

#### Water quality index (WQI)

The water quality index (WQI) is a collective numerical assessment for the overall suitability of water for drinking purposes [10, 35, 63].

In this study, WQI was evaluated because people around the study area rely on these sources of water for consumption. The computation of WQI in this study was based on assigning different weights (W_i_) to water quality parameters according to their influence on the overall quality of water (Table 2).

**Table 2:** Standards (S_i_), assigned weight (W_i_), and weight (W_r_)

Parameter	Standard [30,42]	Weight (W_i_)	Relative weight (W_r_)
EC	1000	5	0.1563
pH	8.5	4	0.1250
DO	5	4	0.1250
Total hardness	150	2	0.0625
Na^+^	200	1	0.0313
K^+^	12	1	0.0313
Ca^2+^	75	1	0.0313
Mg^2+^	100	1	0.0313
Cl^−^	250	4	0.1250
HCO_3¯_	600	2	0.0625
SO_4_	200	2	0.0625
NO^−^_3_	50	5	0.1563
Total		32	1

Secondly, the relative weight (W_r_) for each parameter is calculated using Eq. 1. In this study the following parameters were selected: EC, pH, DO, TH, Na^+^, K^+^, Ca^2+^, Mg^2+^, Cl^−^, HCO_3_^2−^, SO_4_^2−^ and NO_3_^−^ Each of these parameters is assigned a weight Wi ranging from 1 to 5 [14] according to their relative importance in water quality for human consumption and its possible effects on health. In this study, the concentration of these parameters was compared to the standards for drinking water as recommended by WHO [81] and the Standard Organization of Nigeria [73] and was included for the calculation of WQI. Each parameter was calculated as follows:

2 $${\text{W}}_{{\text{i}}} \, = \,{\text{W}}/\Sigma {\text{W}}_{{\text{i}}}$$

W = assigned weight of each parameter.

Wi = sum of assigned weights of all the parameters and the number of parameters.

In this study the following parameters were selected: EC, pH, DO, TH, Na^+^, K^+^, Ca^2+^, Mg^2+^, Cl^−^, HCO_3_^2−^, SO_4_^2−^ and NO_3_^−^ Each of these parameters was assigned a weight Wi ranging from 1 to 5 (Table 2) according to their relative importance for human consumption. The water quality rating (Q_i_) was calculated as:

3 $${\text{Q}}_{{\text{i}}} \, = \,\left( {{\text{C}}_{{\text{i}}} /{\text{S}}_{{\text{i}}} } \right)\,*\,{1}00$$

C_i_ represents the concentration of the parameters and S_i_ is the water quality standard as recommended by [82] for drinking. Finally, sub-indices (SI_i_) and WQI are computed using Eqs. 3 and 4:

4 $${\text{SI}}_{{\text{i}}} \, = \,{\text{W}}_{{\text{r}}} *{\text{Q}}_{{\text{i}}}$$

5 $${\text{WQI}}\, = \,\Sigma {\text{SI}}_{{\text{i}}}$$

The WQI was categorized based on the classification scheme of Batabyal and Chakrabarty [14] as < 50 suitable, 50–100 good, 100–200 poor, 200–300 very poor, and > 300 unsuitable.

#### Contamination level

To determine the contamination level of metal(loid)s in the water resources of the study area, contamination factor (Cf) and contamination index (C_d_) were used to evaluate water quality by calculating the contamination level. In this study, the pollution status of metals (loids) in water was evaluated using the contamination factor (Cfi) as in [25],

6 $${\text{C}}^{{\text{i}}}_{{\text{f}}} \, = \,{\text{C}}^{{\text{i}}}_{{\text{o}}} - {\text{i}}/{\text{C}}^{{\text{i}}}_{{\text{n}}}$$

where C^i^_f_  = c ontamination factor; C^i^_o_–i is the concentration of the element in the sample;

C^i^_n_ = background concentration/maximum allowable limits. Calculated values ​​are classified as low (Cd < 1), medium (Cd = 1–3) and high (Cd > 3) pollution.

#### Irrigation water quality

There are many indicators for monitoring the quality of water for agricultural activities. Some of the parameters used in this study include electrical conductivity (EC), sodium absorption rate (SAR), percentage sodium (%Na), and residual sodium carbonate (RSC). These parameters describe the strength of water alkalinization and its potential impact on soil [57, 67]. The equations for computing these indices are presented as Eqs. 7, 8, and 9: Generally, the alkalinity risk proposed by Richards [66] is expressed as the SAR and it is calculated as:

7 $${\text{SAR}}\, = \,{\text{Na}}^{ + } /\,\sqrt {\left( {{\text{Ca}}^{{{2} + }} + {\text{Mg}}^{{{2} + }} } \right)/{2}}$$

SAR is classified as: excellent, S_1_ (SAR < 10), good, S_2_ (10–18), doubtful,

S_3_ (SAR, 18–26), and unsuitable, S_4_ (SAR > 26) [66].

Sodium Percentage (% Na) was calculated as in Eq. 8:

8 $$\% {\text{Na}}\, = \,\left( {{\text{Na}}^{ + } \, + \,{\text{Ca}}^{{{2} + }} } \right)/\left( {{\text{Na}}^{ + } \, + \,{\text{K}}^{ + } \, + \,{\text{Ca}}^{{{2} + }} \, + \,{\text{Mg}}^{{{2} + }} } \right)\,*\,{1}00\left( {{\text{Todd 198}}0} \right)$$

%Na was classified [74] as excellent (%Na < 20), good (20 < %Na < 40), permissible (40 < %Na < 60), doubtful (60 < %Na < 80) and unsuitable (%Na > 80).

The presence of more carbonate and bicarbonate than the amount of calcium and magnesium determines the appropriate irrigation system [62]. RSC was calculated using Eq. 8, where the ionic strength of all concentrations is expressed in meq/l, as suggested by [23].

9 $${\text{RSC}}\, = \,\left( {{\text{HCO}}_{{3}}^{ - } \, + \,{\text{CO}}_{{3}}^{{{2} - }} } \right)\, - \,\left( {{\text{Ca}}^{{{2} + }} \, + \,{\text{Mg}}^{{{2} + }} } \right)$$

RSCs were classified as safe (RSC < 1. 25), and appropriate (1.25 < 2.5) and inappropriate (RSC > 2.5), [23].

#### Water hazard index (WHI)

The WHI has been used to provide an assessment of the overall quality of different types of water used for various purposes. The WHI was calculated using parameters associated with quarrying activities that are known to negatively impact water quality. These include, As, Cd, Co, Cr, Fe, Ni, Pb, Sr, and Zn. It also makes it possible to compare different bodies of water. WHI was calculated as in [51, 50].

10 $${\text{WHI}}\, = \,\sum {\left[ {{\text{As}}/{1}0 + /{\text{Cd}}/{3} + {\text{Co}}/{1}0 + {\text{Cr}}/{5}0 + {\text{Fe}}/{3}00 + {\text{Ni}}/{2}0 + {\text{Pb}}/{1}0 + {\text{Sr}}/{5}0 + {\text{Se}}/{1}0} \right]} /{9}$$

WHI was categorized as < 1 Low impact (LI), 1 < WHI < 3 moderate Impact (MI), 3 < WHI < 5 high Impact (HI), > 5 Very high Impact (VHI).

#### Human health risk assessment

A risk assessment was used to evaluate the non-carcinogenic risk resulting from water consumption (intake) in adults and children. Contaminant concentrations in drinking water, stream water, and groundwater were calculated from mean daily values (ADD) using Eq. 11 (USEPA 1989):

11 $${\text{ADD}}\, = \,{\text{C}}\,{\text{X}}\,{\text{IR}}\,{\text{X}}\,{\text{EF}}\,{\text{X}}\,{\text{ED}}/{\text{BW}}\,{\text{X}}\,{\text{AT}}$$

ADD in mg/L/day, C (Concentration of trace metal (mg/L), IR (Ingestion Rate), 3.3 L/day), EF (Exposure Frequency), 365 days/year [76, 77], ED(Exposure) Duration),30 years [76, 77], BW (Body Weight), 60 kg Wongassuluk et al. [84], and AT (Average Time), 52 years [76, 77] respectively.

Hazard quotient (HQ) was used to estimate the risks of drinking water contaminated with trace metal(loids) as:

12 $${\text{HQ}}\, = \,{\text{ADD}}/{\text{RfD}}$$

RFD indicates the dose of metal that a person can be exposed to in one day of their life without causing adverse health effects [80]. The RfD values for Cd, Co, Fe, Ni, Pb, Se and Zn are 0.01, 0.0003, 0.7, 0.02, 0.0036, 0.005, and 0.3 respectively. The risk of drinking water contaminated water with more than one metal is determined by the Hazard Index (HI), given by the sum of individual HQ as in Eq. 13:

13 $${\text{HI}}\, = \,\sum {{\text{HQ}}_{{({\text{Cd}})}} +_{{({\text{Co}})}} +_{{\left( {{\text{Fe}}} \right)}} +_{{({\text{Ni}})}} +_{{({\text{Pb}})}} +_{{({\text{Se}})}} +_{{({\text{Zn}})}} }$$

HQ/HI > 1 are indication of health risks from drinking contaminated water [44, 51].

## Results and discussion

### Physicochemical parameters

Table 3 contains a summary of physical and chemical water quality parameters alongside control sites. There were no significant differences among the parameters for the sites. Generally, there are significant differences in the level of physical parameters in different water bodies (pond, stream, well and borehole). Temperature, conductivity, pH, DO and TDS are significantly different in the waters except for pH and DO. This variability could be attributed to the inconsistent rates of ecological, geological, or anthropogenic change [43]. The highest temperature (29.9 °C) was recorded in pond water, while the lowest temperature (29.4 °C) was recorded in the stream water body. EC values for all water from quarrying areas, including values are below 1400 μs/cm [73, 81] guidelines for drinking water. A higher EC average value (157 μs/cm) and the lowest average EC value (89.98 μs/cm) was obtained in the pond water at the basement area, while the lowest average EC value (89.98 μs/cm) was found in the stream water. This indicates higher concentrations of dissolved solutes. Low EC values  < 1400 μs/cm in the study area indicate poor/low mineralization of water in the area which can be attributed to low dilution or solubility of minerals in the area [28]. The fact that TDS values (86.5, 45.91, 65. 42, and 75. 87 mg/l) were < 1000 mg/l and were within the recommended limits showed that the water source has low salinity and is fresh. The mean pH of water from the area is an indication of the acidic nature of water and is attributed to the silicate minerals contained in the rocks and generated wastes rock materials scattered at the quarry site releasing silicic acid into the water as well as breakdown/oxidation of pyrites contained in generated dust and waste rock as shown in Eq. 14:

14 $${\text{12FeS}}_{{2}} \, + \,{\text{45O}}_{{2}} \, + \,{\text{34H}}_{{2}} {\text{O}}\, \to \,{4}\left[ {{\text{H}}_{{3}} {\text{OFe}}\,\left( {{\text{SO4}}} \right){2}\, - \,{\text{2Fe}}\,\left( {{\text{OH}}} \right)_{{3}} } \right]\, + \,{\text{16H}}_{{2}} {\text{SO4}}$$

**Table 3:** Summary of physiochemical data of water in the study area

Parameter & unit	Pond				Stream				Well				Borehole				Sw control	Gw control
	Mean	Min	Max	SD	Mean	Min	Max	SD	Mean	Min	Max	SD	Mean	Min	Max	SD		
Temp (^o^C)	29.7	28.4	29.9	0.93	29.4	28	30.2	0.76	29.4	28	31.4	0.96	29.6	28.4	30.4	0.97	28.19	27.8
EC (µScm^−1^)	157.4	55.7	468	127.8	89.98	18.74	120.9	50.78	118.9	18.7	295	94.81	138	55.7	196.6	117.7	146.37	232
pH	4.73	4.14	5.22	0.48	4.59	1.2	4.1	0.3	4.93	4.21	6.21	0.61	4.63	4.28	5.22	0.37	5.77	6.5
DO (mg/l)	2.54	2	4.6	0.81	2.21	11.2	24.05	1.08	2.42	1.05	4.4	1.15	2.33	2	3	0.5	4.75	4.7
THard.(mg/l)	8.9	4.62	18.4	5.07	7.51	10.3	96.9	5.83	10.38	3.21	34.5	8.11	7.87	4.01	18.43	4.08	40.3	78.4
TDS (mg/l)	86.5	36.7	257.4	70.32	45.91	12.9	106.2	28.39	65.42	10.3	162.3	52.15	75.87	30.6	108.1	64.73	321.8	102
HCO3 (mg/l)	82.5	44.9	269.4	62.27	69.53	44.8	89.8	19.55	91.06	44.8	179.6	45.56	63.83	44.9	102.5	20.6	0.0439	0.0415
NO3 (mg/l)	0.45	0.11	1,23	0.39	0.29	0.11	0.81	0.2	0.36	0.05	2.2	0.46	0.49	0.11	1.23	0.39	0.00031	0.00002
Cl (mg/l)	59.09	27	160	41.36	20.36	9	70	18.22	36.53	2	130	34.92	53	27	160	38.38	0.06587	0.0058
SO4 (mg/l)	14.5	0	38.96	24.8	2.13	1.11	7.38	1.94	13.01	0	57.9	25.4	7.64	0	38.96	12.59	0.01221	0.0042
Na (mg/l)	0.09	0.01	0.23	0.06	0.05	0.02	0.09	0.02	0.04	0	0.1	0.03	0.1	0.05	0.23	0.05	0.00004	0.00002
Mg (mg/l)	0.02	0.01	0.1	0.01	0.01	0.01	0.02	0.01	0.02	0	0.09	0.02	0.02	0.01	0.03	0.01	0.00004	0.00002
K (mg/l)	0.04	0.01	0.13	0.03	0.02	0.01	0.04	0.01	0.02	0	0.05	0.01	0.05	0.01	0.13	0.03	0.00002	0.00001
Ca (mg/l)	0.18	0.03	1.15	0.31	0.08	0.01	0.08	0.07	0.18	0	0.1	0.02	0.08	0.03	0.16	0.06	0.00021	0.00016
Si (mg/l)	0.12	0.04	0.23	0.08	0.07	0.01	0.12	0.05	0.08	0.01	0.21	0.05	0.12	0.04	0.14	0.08	0.02	0
Se (mg/l)	0.19	0	0.14	0.17	0.24	0.04	0.52	0.16	0.21	0	0.52	0.15	0.2	0	0.5	0.17	0.02	0
Fe (mg/l)	0.15	0.02	0.4	0.13	0.16	0.02	0.17	0.15	0.15	0	0.62	0.17	0.14	0	0.34	0.14	0.13	0.12
Mo(mg/l)	0.01	0	0.011	0.001	0.02	0.001	0.004	0.01	0.02	0	0.004	0.002	0.002	0.002	0.11	0.003	0	0
Sr (mg/l)	0.03	0.01	0.05	0.01	0.02	0.01	0.04	0.01	0.02	0	0.05	0.01	0.03	0.01	0.05	0.01	0.22	0
As (mg/l)	0.87	0.23	2.07	0.49	0.37	0.02	0.44	0.37	0.73	0	3.14	0.94	0.6	0.25	0.94	0.25	0	0
Co (mg/l)	0.02	0	0.04	0.01	0.01	0.01	0.04	0.01	0.01	0	0.04	0.01	0.02	0	0.03	0.01	0.01	0
Ni (mg/l)	0.01	0	0.03	0.01	0.01	0.01	0.02	0.01	0.01	0	0	0.01	0.01	0	0.02	0.01	0.02	0
Zn (mg/l)	0.05	0.02	0.21	0.05	0.04	0.02	0.03	0.02	0.04	0	0.11	0.03	0.03	0.01	0.05	0.01	0.057	0.05
Pb (mg/l)	0.42	0.12	1.28	0.38	0.12	0.01	0.12	0.11	0.15	0.07	0.41	0.1	0.44	0.02	1.28	0.39	0.053	0
Sb(mg/l)	0.005	0	0.02	0.005	0.004	0	0.01	0.03	0.003	0.0001	0.04	0.12	0.003	0.001	0.06	0.001	0	0
Ag (mg/l)	0.07	0	0.11	0.09	0.03	0.01	0.07	0.03	0.07	0.01	0.82	0.18	0.05	0	0.14	0.04	0	0
Cd (mg/l)	0.24	0.02	0.31	0.23	0.03	0.01	0.09	0.01	0	0	0	0	0.23	0.02	0.81	0.24	0	0
Mn (mg/l)	0	0	0	0	0.01	0.01	0.01	0.01	0	0	0	0	0.01	0	0.01	0.01	0	0
Cu (mg/l)	0.03	0.01	0.81	0.02	0.03	0.01	0.04	0.01	0.02	0	0.02	0.01	0.04	0.02	0.12	0.05	0.03	0.01
Cr (mg/l)	0.34	0.01	0.36	0.75	0.36	0.02	1.02	0.01	0.04	0.01	0.03	0.1	0.01	0	0.04	0.02	0.03667	0.01

Additional source of acidity may be as result of the humic acids coming from organic matter [10]. Acidic water influences the release of metals into the water bodies [49]. The highest mean pH value (4.93) was obtained from hand dug-well water samples of the quarry site in the sedimentary area with the lowest value (4.59) obtained in the stream water samples of the basement area. The higher pH value from the sedimentary area could be a result of the acid-neutralizing capacity linked with the presence of the carbonates from limestone of the quarry site in the area [85].

The mean values of DO (2.54 mg/l, 2.21 mg/l, 2.42 mg/l and 2.33 mg/l) for all the waters samples from the quarry sites in the basement area and that from the Calabar flank area are all below the recommended [81] guideline of 5.0 mg/l for drinking water. Ellis [29] stated that the amount of dissolved oxygen in each water sample is determined by the balance between biological oxygen production and consumption. The low DO content in water in the study area can be attributed to the high temperature in the area, which reduces the DO content. Additionally, Ellis [29] stated that at high temperatures, water tends to release gas into the air, resulting in dissolved oxygen (DO). The mean and range of values of total hardness values in all water samples are indicative of the softness of the water in the area (Total hardness (TH) < 75 mg/L).

The main cations (Na^+^, K^+^, Ca^2+^ and Mg^2+^) and anions (HCO_3_^−^, Cl^−^, SO_4_^2−^ and NO_3_^−^) found in the waters of the study area are shown in Tables 3 and 4 with Ca^2+^, and HCO_3_^−^ as the main cations and anions, respectively, in waters taken from all examined sites. The abundance of major anions has shown that bicarbonate (HCO_3_^−^) accounts for 60% of the total anions in all water sources in respective of the geologic controls. The amount of carbonates and bicarbonates in groundwater may be responsible for the decomposition of carbonates and the dissolution of carbonic acid due to chemical weathering [19, 37, 41, 70]. The order of abundance of anions among the water types, active and abandoned sites is HCO_3_^−^ > Cl^−^ > SO_4_^2−^ > NO_3_. Chlorides are the second most abundant anion in the water resources. Chloride ion (Cl^−^) recorded average concentrations of 59.09, 20.36, 36.53, and 7.64 mg/l in ponds, streams, boreholes, and well water from the quarry sites were below the allowable limit values of 250 mg/L for Cl^−^ and do not seem to pose any environmental problem. Chloride can be used as an advance warning of the presence of other toxic contaminants [83]. The sulfate (SO_4_^2−^) value of all water samples is below 250 mg/l, which is the acceptable limit in water. Besides quarry waste, other sources of chloride, and sulphate can come from rock water interaction, rainwater, industrial fertilizers such as gypsum compost, sewage, municipal waste, and leachate seepage [13] from quarry tailings and rock water interaction [79] However, when sulfates and chlorides accumulate on the water in the human body, it can cause heartburn, high blood pressure, dehydration, asthma, and osteoporosis [32].

**Table 4:** Varimax principal component analysis (PCA) of all water resources grouped together for physicochemical parameters

Parameter	Factor				
	1	2	3	4	5
Temp (^o^C)	0.15	0.05	−0.01	−**0.55**	−0.43
pH	**0.74**	0.10	−0.02	0.01	−0.24
EC (μS/cm)	**0.93**	0.06	0.02	0.05	−0.08
TDS (mg/l)	**0.93**	0.06	0.02	0.05	−0.08
DO	0.39	−0.25	0.40	0.08	−0.35
TH	0.37	−0.17	**0.73**	0.23	−0.11
Na^+^	−0.17	**0.71**	−0.10	−0.45	0.24
K^+^	−0.16	0.54	−0.19	−0.44	0.44
Ca^2+^	**0.96**	−0.05	0.00	0.09	−0.01
Mg^2+^	**0.89**	−0.19	0.01	−0.02	0.01
Cl^−^	**0.92**	0.02	0.16	−0.08	−0.02
HCO_3¯_	**0.90**	−0.17	−0.03	0.09	0.09
SO_4_^2^_¯_	**0.95**	−0.05	0.09	0.05	−0.16
NO^−^_3_	−0.13	0.02	0.46	−0.08	0.14
Ag (μg/l)	−0.23	0.09	−0.46	0.34	−0.02
As	−0.33	−0.11	−0.27	−0.20	0.44
Cd	0.17	−0.44	−0.45	0.03	0.31
Co	0.42	−0.29	0.09	0.18	−0.08
Cr	−0.22	0.09	0.18	−0.17	**0.57**
Cu	−0.04	0.02	0.21	−**0.82**	0.09
Fe	0.05	0.15	0.02	0.04	**0.84**
Mo	−0.19	0.00	−0.45	0.10	0.30
Mn	0.07	0.45	−0.04	0.12	−0.38
Ni	**0.76**	−0.40	−0.03	−0.13	0.06
Pb	0.04	−**0.64**	−0.10	−0.32	0.10
Sb	0.17	**0.61**	0.05	0.08	0.32
Se	0.02	−0.08	−**0.74**	0.18	−0.05
Si	−0.18	0.07	**0.62**	−0.19	0.42
Sr Zn	**0.81**	0.18	0.02	0.07	0.08
	−0.08	0.01	−0.03	−**0.89**	−0.01
Eigen value	9.14	3.22	2.67	2.18	1.98
% Total variance	30.47	10.72	8.90	7.27	6.61
Cum eigen value	9.14	12.36	15.03	17.21	19.19
Cum % total variance	30.47	41.19	50.09	57.36	63.97

The lowest concentration of major anions in the study area is nitrate (NO^−^_3_) with mean concentrations of 0.45, 0.29, 0.36, and 0.49 mg/l for pond, stream, borehole and well water samples from the quarry sites. The concentration of NO_3_^−^ is generally below the maximum allowable limit [73, 81] in all the water samples. Nitrate is the product of atmospheric fixation of organic nitrogen and a byproduct of nitrogen transformation [41]. Chloride, nitrate, and sulfate are considered indicators of anthropogenic pollution.

For the major cations, calcium is the dominant ion, and accounts for over 65%, while Na ions account for over 17% and Mg and K ions account for about 10.2, and 7.8% respectively. The order of abundance is Ca^2+^ > Na^+^ > Mg^2+^ > K^+^. The mean values of the cations in the water samples were within the WHO [81] and SON [73] permissible limits of 75, 200, 100, and 12 for Ca^2+^, Na^+^, Mg^2+^, and K^+^ respectively. According to WHO [81] high concentrations of calcium and sodium above the admissible level may cause health problems such as kidney stones, abnormal nervous systems, and cardiovascular diseases such as high blood pressure. The main source of Ca^2+^ in water is the dissolution of carbonates from sedimentary rocks and minerals like calcite, dolomite, and limestone. Moreover, agricultural activities contribute significantly to the release of calcium and magnesium through weathering processes of silicate minerals and hydrolysis of CaCO_3_^−^ and Ca-Mg-(CO_3_)^2−^, both of which are magnesium-rich minerals [11].

The interaction between calcium and magnesium is very important in the type of water in terms of hardness. Most of the different types of water in the study area contain more calcium than magnesium. Differences in water hardness in the study area may be due to the relative levels of Ca-Mg-HCO3^−^ ions in the solution. Comparative analysis of cations and anions in ponds, streams, hand dug wells and freshwater from quarries in the Oban massif and Calabar showed that the most important ions in the study were Ca and HCO_3_^−^.

Saturation indices of mineral phases determined revealed that the water resources are oversaturated with, carbonates, goethite, and hematite with positive S.I values above zero and undersaturated with halite, anhydrite, aragonite, calcite, dolomite, gypsum, quartz, sylvite, talc having values below zero.

Associated with the major anions and cations are minor metal(loids) which are also constituents of water and usually present potential health risks to aquatic organisms and humans. High temperatures and low pH values enable this metal(loid)s to be easily released into the water [20]. Long-term exposure to polluted water for a long period can affect the normal functioning of the human body, as it interacts with biological molecules containing nitrogen, oxygen, and sulfur, triggering structural and functional changes [16].

**Fig. 4:**
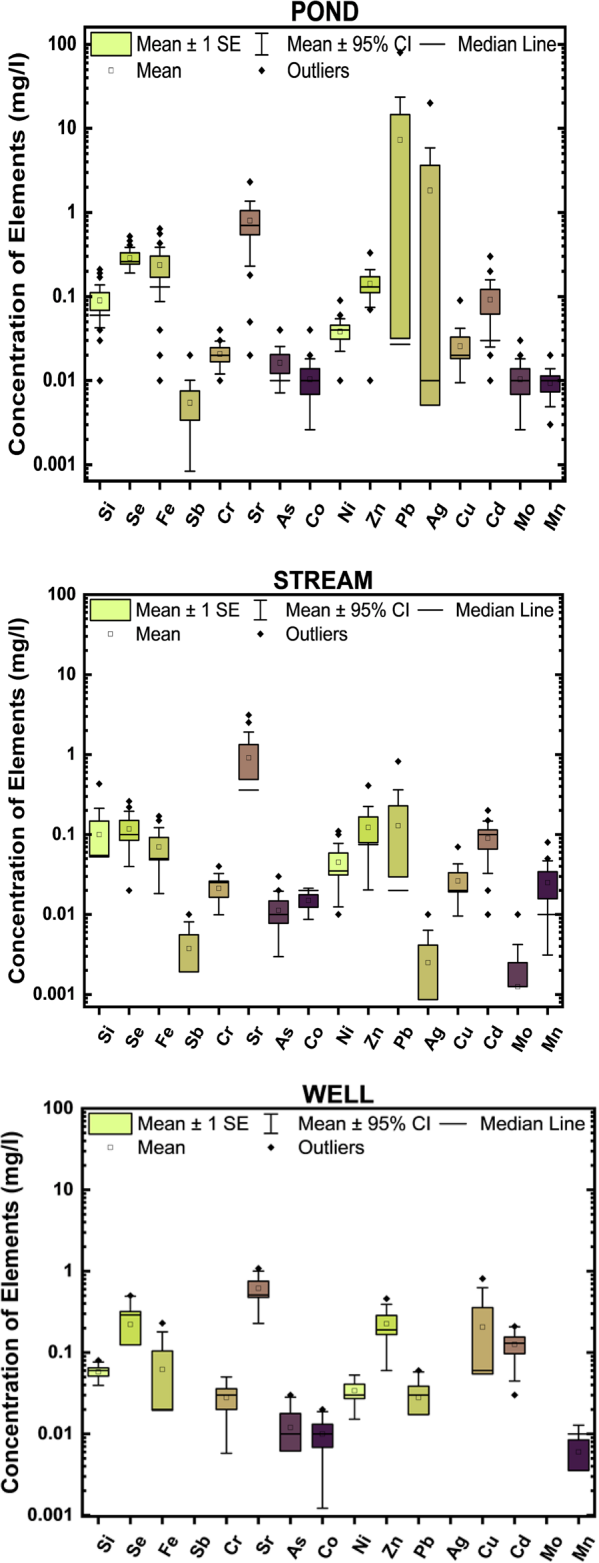

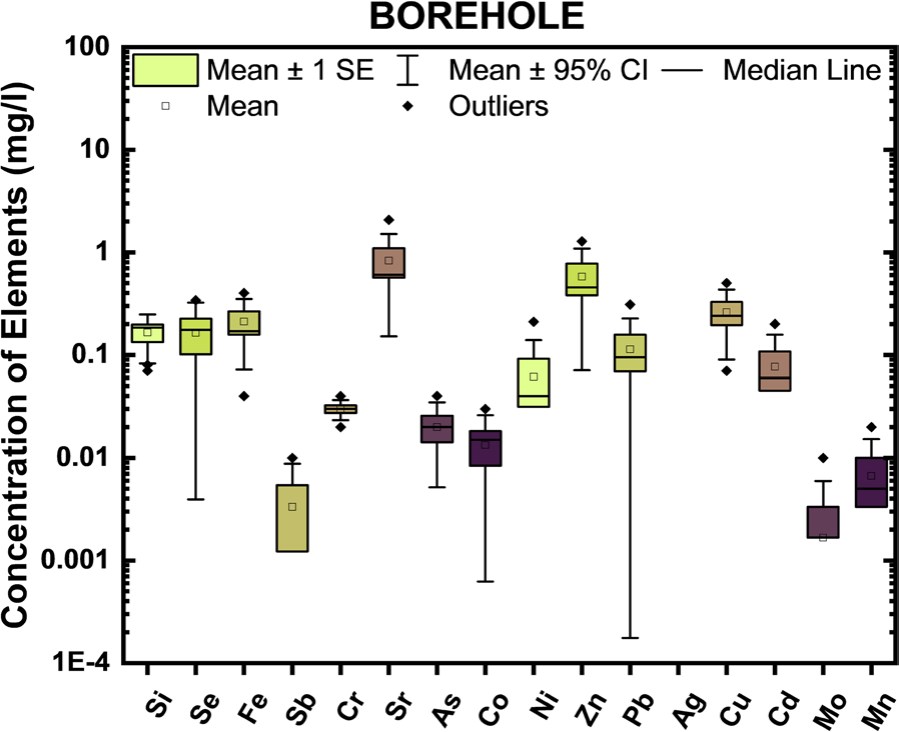
Box and whisker plots of metal (loid) in Pond, stream, well and borehole

The spatial variation of concentration of metal(loids) is presented as box and whisker plots (Fig. 4). The box plot is a powerful statistical tool that shows the distribution data across means, ranges, median, and range. Since the distributions of water quality parameters are  often skewed to the right, it is preferable to consider the median as the indicator of the overall trend. The box’s centred on the horizontal line, the 25th and 75th percentiles (quartiles), on the top and bottom of the box [8]. Figure 4 shows distinct differences and variations of the metal(loid)s with no defined pattern. The data set did not fit a log-normal distribution.

Table 3 and Fig. 4 showed that the large variation and standard deviation in the parameters indicate that the geochemical properties of water resources are affected by various processes, which may be the interaction of rock weathering with anthropogenic activities [71]. The average concentration (Table 3) of the metals and metalloids decreased as As > Pb > Cr > Cd > Se > Fe > Si > Ag > Zn > Ag > Cu = Sr > Co > Mn = Ni > Sb. Mean concentration of all parameters in the active and abandoned sites are within the [73, 81] standard limits except for Se, Ni, Pb, Cd, Sb in the active sites and Se, Cr, Ni, Pb in the abandoned sites. The result of this gives indication of the enrichment of these metals in the area. In the study area there is no defined pattern of distribution of the metal(loidd)s. The trend of dominance in the composition of trace metals from the active quarry sites in the Oban Massif is in the order Sr > Cr > Zn > Se > Si > Pb > Ni > Cu > As ≥ Cd = Mn > Sb. The trend of dominance from the abandoned sites in Oban Massif is in the order Sr > Cr > Se > Zn > Si > Pb > Ni = Mn > As = Cu > Sb > Cd while the trend of dominance from the active quarry site in Calabar Flank is Sr > Ni > Se > Zn > Pb > Si > Cu > Co > Cr respectively. Similarly, comparison of metals among different water types shows that the trend of dominance of metals in water is in the order Sr > Se > Zn = Fe > Ni > Si > Pb = Co > Cu > As = Sb = Cr = Mn(pond),Sr > Se > Zn > Fe > Pb > Si > Ni > Cu = Cr > As = Co = Ag(stream),Sr > Pb > Ni > Fe > Zn > Cu > Se > Si > Cr > Cd = Mn(borehole),andSr > Zn >  > Cu > Se > Fe > Si > Pb > Ni > Cr > As = Co(well).

Pond water recorded about 67% of these metals from the basement and sedimentary area. This could a result of higher anthropogenic inputs in water from these sites. The occurrences of Si may be attributed to the dissolution of silicate-bearing bedrocks of the study area. The presence of an alkaline environment and the weathering of silicate minerals in these rocks have also been mentioned as sources of silica in water by [71]. Similarly, the metal content in borehole water samples(B6) from the sedimentary area was seen to be higher than those from the well water samples of the basement area, although samples from these two sources could be comparable. The reason for this difference may be due to lithological differences in the study area. The total metal content in pond and stream water samples was higher than the samples obtained from hand-drilled wells and boreholes and this may be due to high anthropogenic contamination load to the pond and stream waters*.* In all cases, other than agricultural activities and mining, apparent high values can be mainly due to geogenic activities and activities associated with quarrying through waste, scrap and emissions from vehicles and machinery.

### Principal Component Analysis (PCA),

**Fig. 5:**
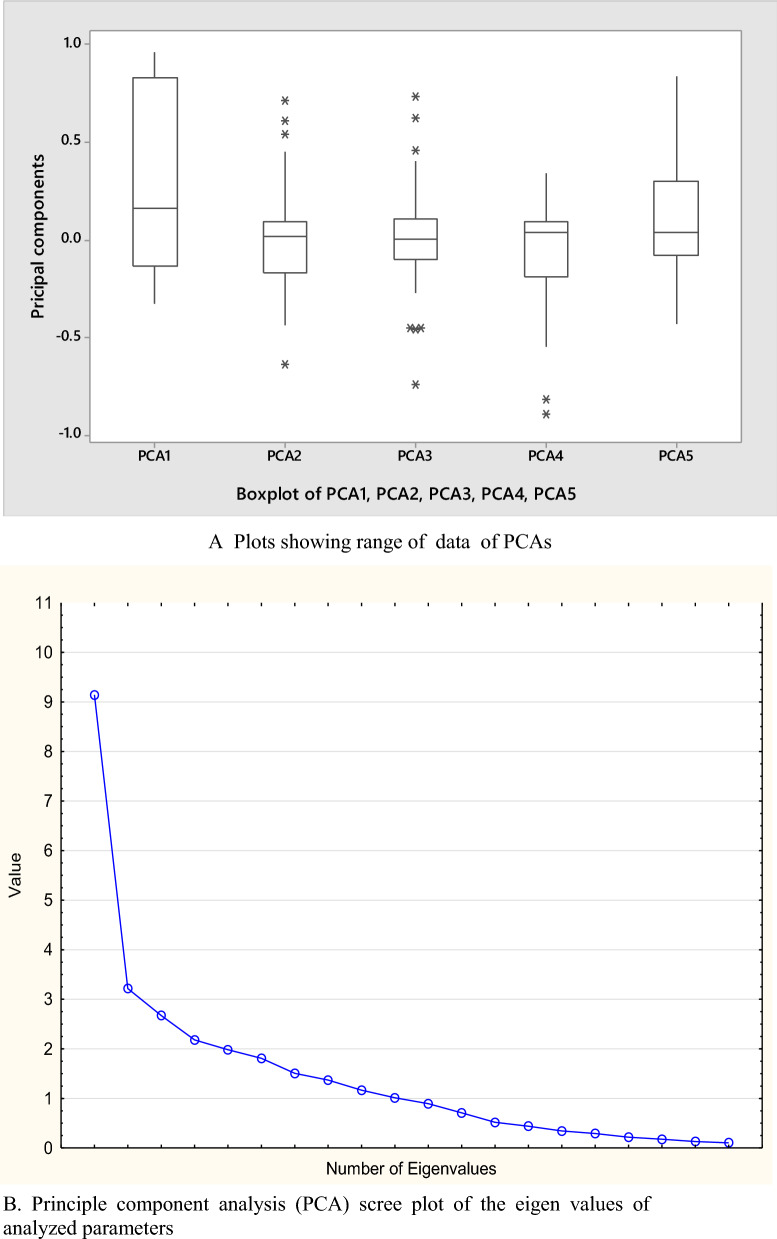
Box plot showing range of PCAs (**A**) and Scree plot (**B**) of the eigen values of analyzed parameters

A Principal component analysis (PCA) was performed for the various water resources grouped using the physicochemical parameters. The principal component analysis with varimax rotation eigenvalues  > 1 is shown in Table 4 as the variance of the PCAs (Fig. 5a). PCA 1 showed a variance of 30.47% with higher weights for pH, TDS, EC, Ca^2+^, Mg^2^ + , Cl^−^, HCO_3_^−^, SO_4_^2−^, Ni, and Sr. This relationship demonstrates the influence of environmental factors on the chemistry of the water. Positive loading on Na^+^ K^+^ HCO_3_^−^, Cl^−^, and SO_4_^2−^ has been observed and is associated to indicate the presence of both natural (geogenic) and human (anthropogenic). The natural source is weathering of rocks and minerals into the water [72] while the anthropogenic source includes atmospheric deposition with contributions from the dispersed dust during the quarry operations as well as leaching from waste rock materials scattered at the quarry sites (Fig. 1a, b) into the surface water (pond and stream). High loadings for HCO_3_^−^, Ca^2+^, and Mg^2+^ can be attributed to the natural weathering of silicate minerals from the basement and sedimentary rocks and this has been reported as the main source of these ions by [70]. Additionally, high loading for Cl^−^, and SO_4_^2−^ indicate that they were added from recharging rainwater, while the high loading on Ni, and Sr was due to minor variations in lithology. The high positive loading of HCO_3_^−^ was due to atmospheric CO_2_ combined with soil water and carbonate dissolution [49]. PCA 2 explained 10.72% of the total variance with the highest loading for Na and Sb, and the athitectic relation with Pb suggest their different sources. The positive loading value of hardness is attributable to natural elements of silicate rocks such as pyroxene, biotite etc. PCA 3 accounts for 8.9% of total variance with high positive loadings for hardness and Si and negative loading on Se indicating their different sources. PCA 4, has a 7.27% total variance with negative loading for temperature, Cu and Zn depicting that the concentration of Cu and Zn are not influenced by temperature and are of mixed sources. PCA 5 accounts for 6.61% of variance with high loadings for Cr and Fe reflecting their geogenic sources and water–rock interactions leading to rock disintegrations. In general, the PCA did not show significant correlations between metals and metalloids in the aqueous phase. This may be due to the low mineralization of water in the area which reflects of low solubility of the minerals in the water. A graphical representation (Fig. 5b) has been used to identify the principal components that could be retained and to describe the variability of the original dataset, suggesting that the five components account for 67.97% percent of the total variance. As a result, the details of the water quality at each of the sampling locations may be calculated using only five variables.

### Hydro-chemical facies and water types

**Fig. 6:**
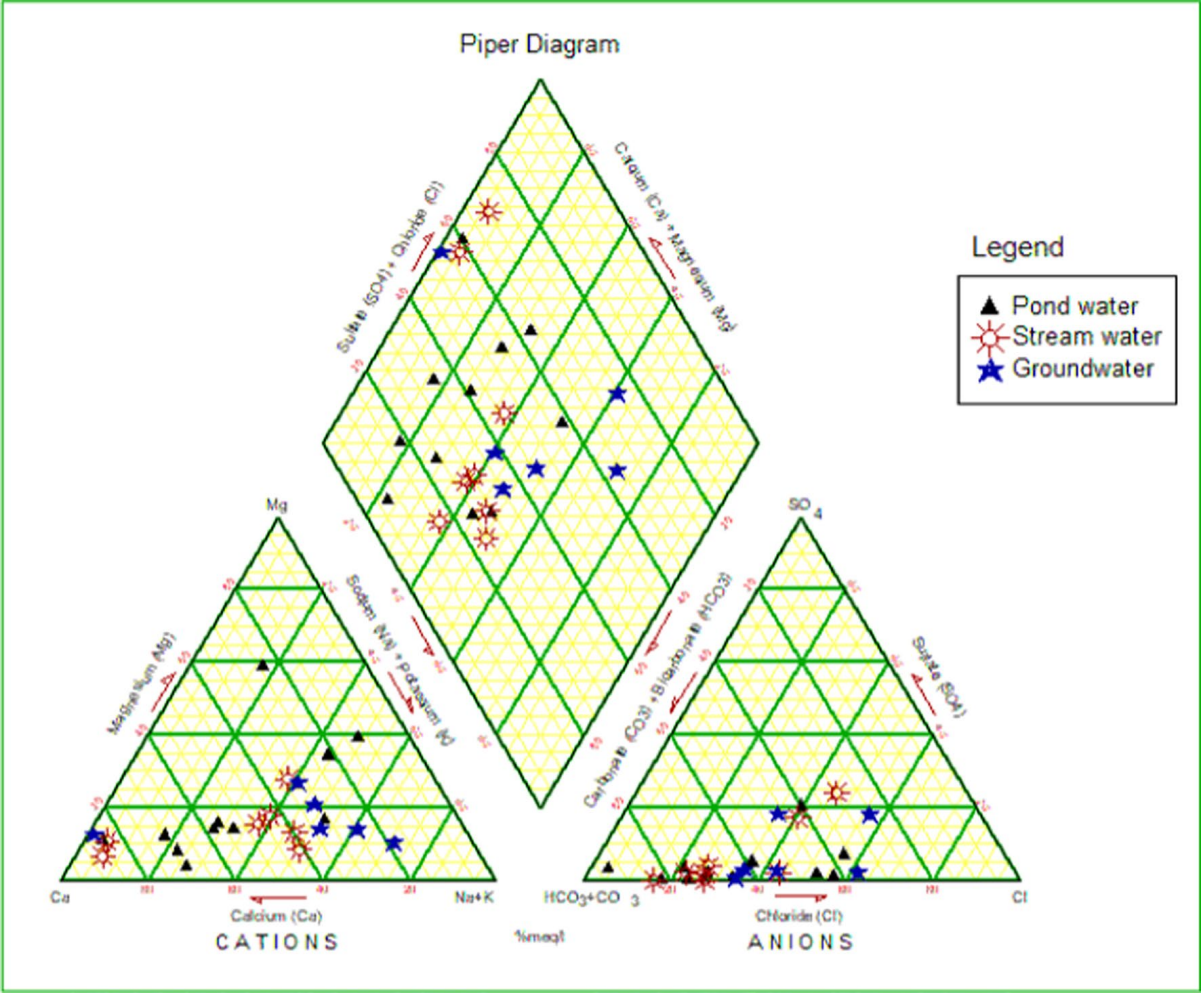
Pipers Diagram showing hydrogeochemical character of pond, stream and groundwater

**Fig. 7:**
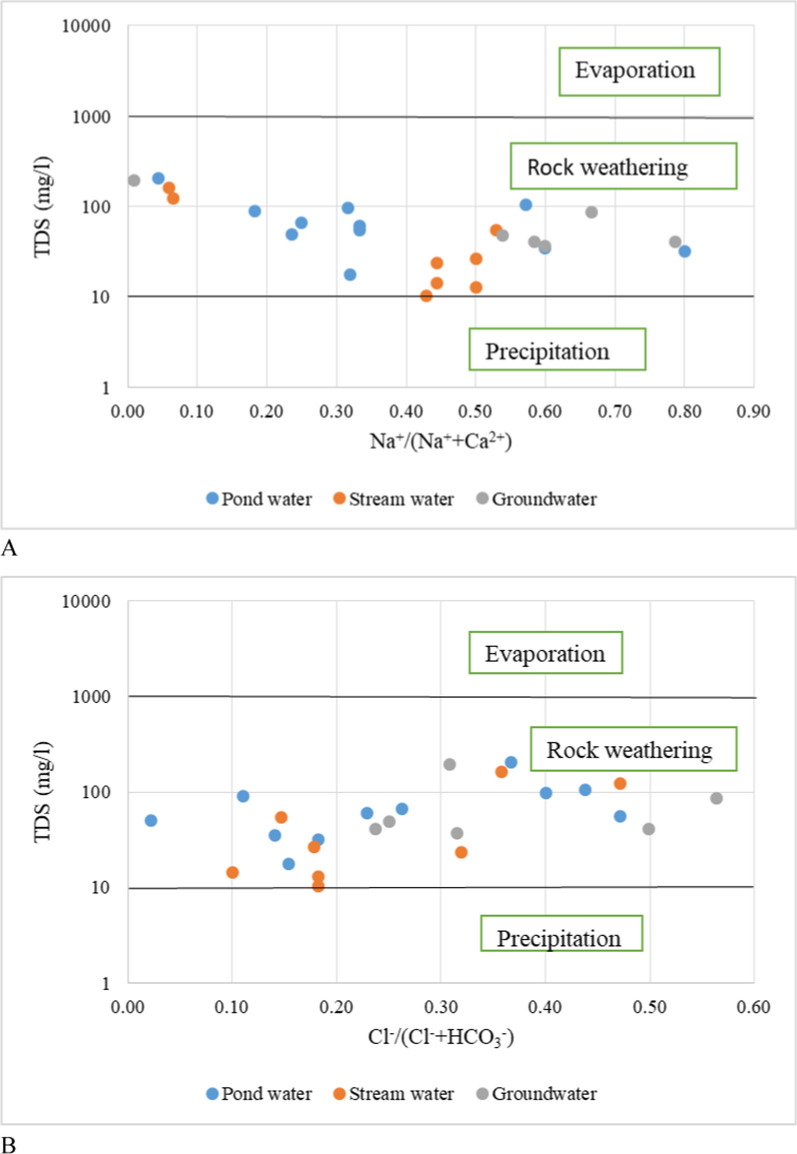
Gibbs Diagram of **A** TDS versus Na^+^/(Na^+^ + Ca^2+^), **B** TDS versus Cl^−^/(Cl.^−^ + HCO_3_

**Fig. 8:**
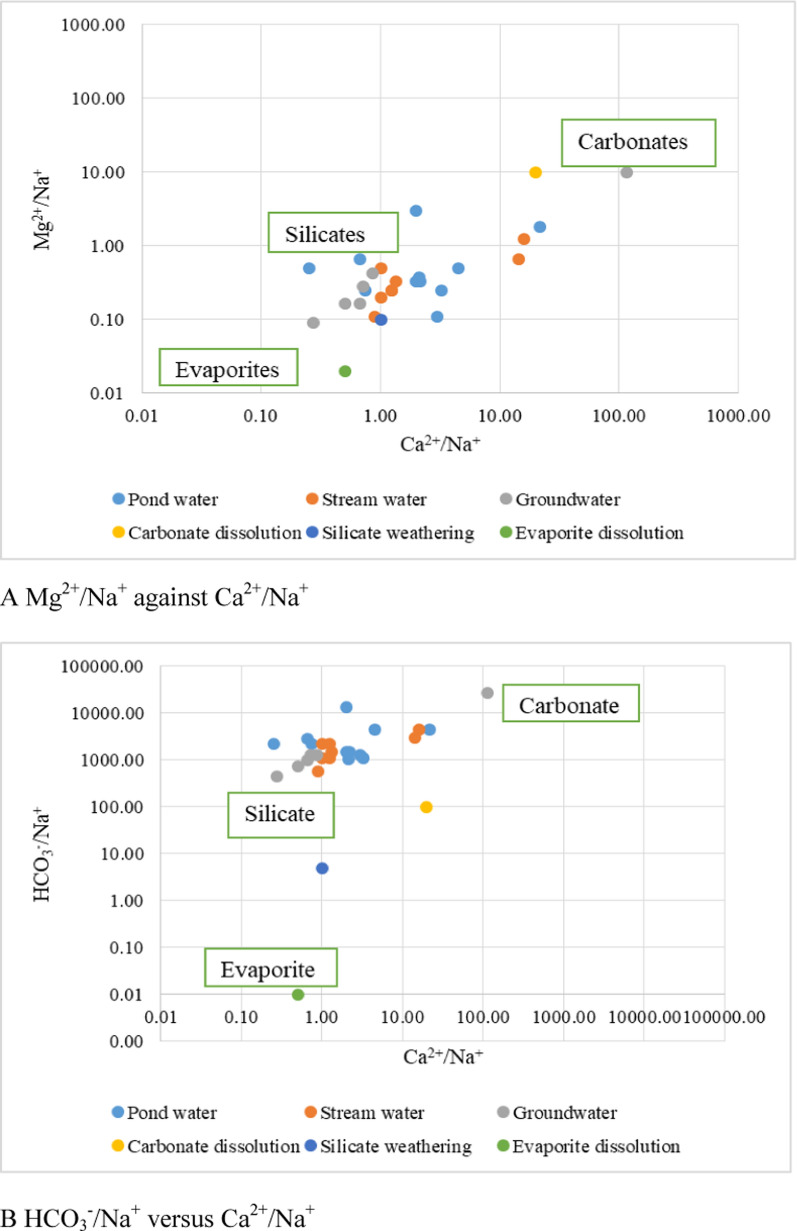
Cross plot to distinguish the sources of major ions

In this study hand drilled wells and boreholes were evaluated as groundwater. All the water samples collected from the different water sources from both quarries revealed two major water types: Ca-Mg-SO_4_-Cl and Na–K-HCO_3_ (Fig. 6, Appendix 1). These water types probably result from the disintegrations of feldspars and carbonate rocks, and changes in the lithology in the study area. In addition, the presence of Na–K-HCO_3_ may indicate the effect of the combined effects of cations exchange and calcite/carbonate or silicate dissolution [9]. According to [47, 71], bicarbonates and calcium in groundwater samples could be due to the natural dissolution of carbonate minerals. Water chemistry is controlled by many processes including rainfall recharge, hydrological interactions, water–rock interaction, and human activities [17, 70]. Gibbs plot (Fig. 7) and cross plots (Fig. 8) of Mg^2+^/Na^+^ versus Ca^2+^/Na^+^ and HCO_3_^−^/Na^+^ versus Ca^2+^/Na^+^ to distinguish the sources of ions in water resources of the study area confirm rock/silicate weathering as the main processes controlling the chemistry of the water resources in the quarry areas. Weathering of alumina-silicates minerals [70] are the major contributor of Na^+^, K^+^, Ca^2+^, Mg^2+^, and HCO_3_^−^. The effect of weathering activities in the study area is that it increases the dissolution of silicates and carbonates, as well as ion exchange in water. Pyroxene, Ca-plagioclase, orthoclase, amphiboles, olivine, and biotite are the main silicate minerals found in the crystalline rocks of the region (Appendix 2). The general reaction for the weathering of silicate rocks is shown in Eq. 15:

15 $$\left( {{\text{Na}},\,{\text{Ca}},\,{\text{Mg}},\,{\text{K}}} \right)\,{\text{silicates}}\, + \,{\text{H}}_{{2}} {\text{CO}}_{{4}} \, \to \,{\text{H}}_{{4}} {\text{SiO}}_{{4}} \, + \,{\text{HCO}}_{{3}} \, + \,{\text{Na}}\, + \,{\text{Ca}}\, + \,{\text{Mg}}\, + \,{\text{K}}\, + \,{\text{Clay}}\,{\text{minerals}}$$

The Na and K, Ca and Mg may be attributed to cation exchange process [42].

The incongruent dissolution of plagioclase in water can be written as:

16 $${\text{4Ca}}_{{0.{5}}} {\text{2Na}}_{{0.{5}}} {\text{Al}}_{{2}} {\text{Si}}_{{2}} {\text{O}}_{{8}} \left( {\text{s}} \right)\, + \,{\text{6CO}}_{{2}} \, + \,{\text{9H}}_{{2}} {\text{O}}\, = \,{\text{3Al}}_{{2}} {\text{Si}}_{{2}} {\text{O}}_{{5}} \left( {{\text{OH}}} \right)_{{4}} \, + \,{\text{2Na}}\, + \,{\text{2Ca}}\, + \,{\text{6HCO}}_{{3}} \, + \,{\text{4SiO}}_{{2}}$$

For olivine, it is given as:

17 $${\text{Mg}}_{{2}} {\text{SiO}}_{{4}} \left( {\text{s}} \right)\, + \,{\text{4H}}_{{2}} {\text{CO}}_{{3}} \, = \,{\text{2Mg}}\, + \,{\text{4HCO}}_{{3}} \, + \,{\text{H}}_{{4}} {\text{SiO}}_{{4}}$$

The weathering activities in this study imply the effect of dissolution of minerals such as plagioclase, amphiboles, calcite among others with the release of ions and metals into the environment.

### Assessment of water quality

**Fig. 9:**
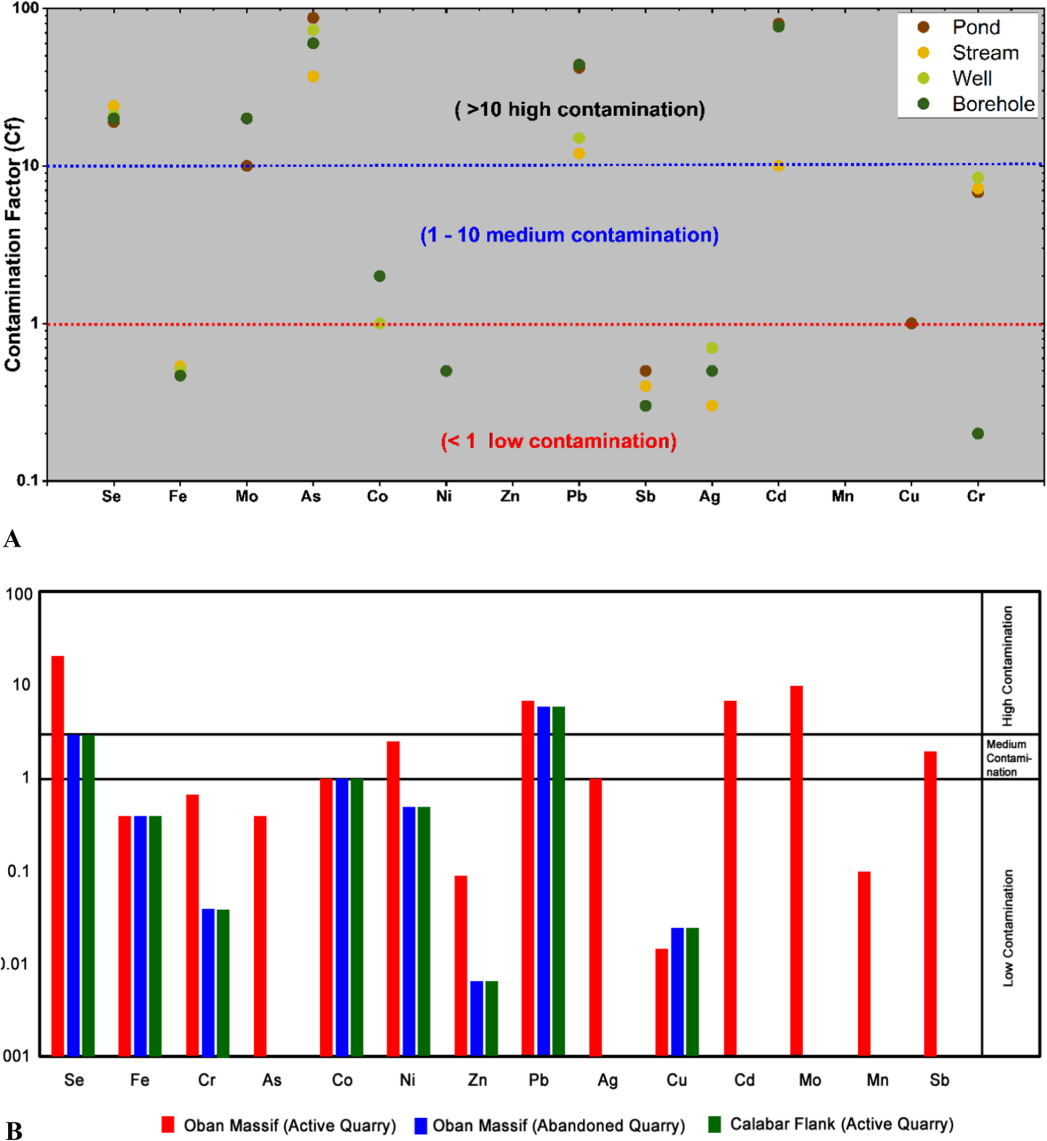
**a** Contamination class of metal(loid)s of the different water resources in the study area. **b** Calculated Contamination factor (Cf) of metal (loids)

**Fig. 10:**
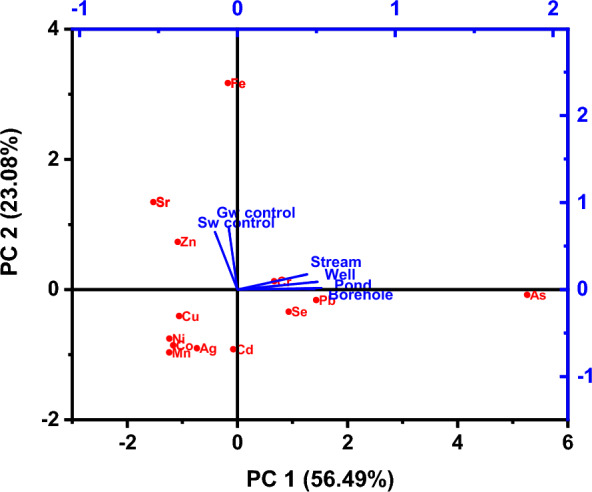
Boxplots showing variation of the metal(loids) in the various water resources

The assessment of water quality in this study was carried out to determine the portability of water for drinking and other domestic uses. This was done based on a comparison of the analytical results obtained from the quarry sites existing water quality guidelines [73, 81]. Based on the analyzed data for P, S, W, and B, 100% of pH and DO were not within the [73, 81], maximum admissible level. The mean concentration of As, Cd, Pb, and Se for all the water sources were above that of the WHO [81] and SON [73] admissible limits of 0.01 mg/l (As, Pb, and Se) and 0.003 mg/l(Cd) suggesting that the water resources are not suitable for drinking and irrigation for these parameters. The computed WQI varied from 11.27 to 37.26 (Table 5) showing that the water resources are suitable (WQI < 50) for drinking as classified by Batabyal and Chakrabarty [14] and are in A grade class. The mean contamination factor of the metal(loid)s (Fig. 9a and Appendix 1) has shown that 50%(Pond), 42%(Stream), 35.7%(Well) and 42.86(Borehole) were moderately and highly contaminated with As, Cd, Co, Cr, Mo, Pb and Se. These metal(loid)s are halophilic [45] in nature, have affinity for Sulphur and thus reflect the main potentially toxic elements (PTE) signature indicative of bedrock geology [50]. In respect of the geologic terrain and status of the quarry sites (Fig. 9b), the water was not polluted with Fe, Cr, Co, Cu, Zn., Ag, and Mn but were polluted with Cd, Mo, Pb, and Se. Using the classification scheme, Cd, Mo, Pb, and Se form Oban massif active sites are in the class of high contamination and Ni, and Sb are in the class of medium contamination, whereas Se and Pb from the Oban massif abandoned and Calabar flank active sites are in the class of medium contamination and thus not suitable for consumption based on these parameters. As shown on Fig. 10, Cr is the main contribution of pollution to the stream water, whereas As, Pb, and Se are associated with pond and borehole water which is explained by PCA 2 constituting 56.49% of the variance. Also, Fe is associated with the control area and is explained by PCA 1 which is 23.08% of the total variance. These associations signify areas with a significant concentration, influenced by comparable geological, geochemical, environmental, and anthropogenic sources.

**Table 5:** Water Quality Index (WQI)

Parameter	Code	WQI	Remarks	Grade
		Values		
Pond	P1	14.00	Excellent	A
	P2	13.49	Excellent	A
	P3	16.11	Excellent	A
	P4	12.36	Excellent	A
	P5	17.53	Excellent	A
	P6	17.46	Excellent	A
	P7	22.35	Excellent	A
	P8	19.48	Excellent	A
	P9	19.54	Excellent	A
	P10	21.58	Excellent	A
	P11	31.76	Excellent	A
Stream	S1	12.14	Excellent	A
	S2	13.06	Excellent	A
	S3	11.35	Excellent	A
	S4	19.34	Excellent	A
	S5	11.27	Excellent	A
	S6	22.17	Excellent	A
	S7	29.11	Excellent	A
	S8	33.57	Excellent	A
**Hand dug well**	**W1**	**35.6**	**Excellent**	**A**
	**W2**	**43.2**	**Excellent**	**A**
	**W3**	**21.6**	**Excellent**	**A**
	**W4**	**31.4**	**Excellent**	**A**
	**W5**	**17.8**	**Excellent**	**A**
Borehole	B1	15.22	Excellent	A
	B2	17.55	Excellent	A
	B3	19.13	Excellent	A
	B4	17.82	Excellent	A
	B5	17.40	Excellent	A
	B6	37.26	Excellent	A

#### Suitability for irrigation

Adequate irrigation capacity of ponds, streams, and groundwater for the agricultural needs of the population was evaluated using four parameters including electrical conductivity (EC), sodium percentage (%Na), sodium adsorption rate (SAR), and residual sodium carbonates (RSC). The EC of all the water samples ranged from 18.70 and 372.0 with an average of 122.89 μS/cm (Table 6). All the samples showed EC < 2,250 μS/cm and were considered excellent for irrigation use [66]. However, all the samples from the sedimentary area (P11, S8, and B6) were classified as Good (250 < EC < 750 μS/cm). According to SAR, all the water samples fall into the excellent category (SAR < 10). Regarding %Na (Table 6), 27, 45, and 27% of PW and 25, 50 and 25% of SW are in the excellent category (%Na < 20), good (20 < %Na < 40), and permissible water (40 < %Na < 60) respectively. For GW, 17%, 50% and 33% of the samples were in the excellent, permissible, and doubtful categories (60 < %Na < 80). Similarly, RSC values showed 9.82% and 9% of P and 50. 33% and 17% of GW were safe, marginally suitable, and unsuitable for irrigation (Table 7). For SW, 75%, and 25% were safe and marginally favorable for irrigation. Also, from the sedimentary areas (P11, S8, and B6) were not suitable for irrigation use based on RSC. According to [23], high value of RSC value in water means more sodium adsorption by water (Table 6).

**Table 6:** Irrigation water quality for different water types in the area

Parameter	Code	EC (μS/cm)	Remarks	SAR	Remarks
		Value		Value	
Pond water	P1	63.4	Excellent	0.036	Excellent
	P2	90.7	Excellent	0.041	Excellent
	P3	164	Excellent	0.017	Excellent
	P4	32.5	Excellent	0.047	Excellent
	P5	120.9	Excellent	0.046	Excellent
	P6	110.8	Excellent	0.010	Excellent
	P7	193	Excellent	0.051	Excellent
	P8	176.1	Excellent	0.041	Excellent
	P9	58.6	Excellent	0.053	Excellent
	P10	100	Excellent	0.030	Excellent
	P11	372	Excellent	0.012	Excellent
Stream water	S1	42.9	Excellent	0.043	Excellent
	S2	100	Excellent	0.080	Excellent
	S3	48.4	Excellent	0.053	Excellent
	S4	23.4	Excellent	0.029	Excellent
	S5	18.7	Excellent	0.035	Excellent
	S6	26.1	Excellent	0.043	Excellent
	S7	222	Excellent	0.012	Excellent
	S8	295	Excellent	0.013	Excellent
**Hand dug well**	**W1**	**36.7**	**Excellent**	**0.023**	**Excellent**
	**W2**	**76.15**	**Excellent**	**0.013**	**Excellent**
	**W3**	**84.7**	**Excellent**	**0.015**	**Excellent**
	**W4**	**56.71**	**Excellent**	**0.025**	**Excellent**
	**W5**	**49.58**	**Excellent**	**0.014**	**Excellent**
Borehole	B1	66.7	Excellent	0.069	Excellent
	B2	75.3	Excellent	0.140	Excellent
	B3	158	Excellent	0.076	Excellent
	B4	74.4	Excellent	0.067	Excellent
	B5	88.3	Excellent	0.058	Excellent
	B6	351	Excellent	0.002	Excellent
Max		372.00		0.140	
Min		18.70		0.002	
Mean		122.89	Excellent	0.044	Excellent

**Table 7:** Irrigation water quality for different water types in the area

Parameter	Code	Na%	Remarks	RSC	Remarks
		Value		Value	
Pond water	P1	43.51	Permissible	1.42	Marginally suitable
	P2	22.38	Good	1.43	Marginally suitable
	P3	17.35	Excellent	1.46	Marginally suitable
	P4	27.77	Good	1.36	Marginally suitable
	P5	26.48	Good	1.86	Marginally suitable
	P6	16.36	Excellent	2.24	Marginally suitable
	P7	51.62	Permissible	1.49	Marginally suitable
	P8	27.57	Good	1.49	Marginally suitable
	P9	47.86	Permissible	1.49	Marginally suitable
	P10	32.01	Good	0.74	Safe
	P11	3.76	Excellent	3.67	Not suitable
Stream water	S1	37.37	Good	0.74	Safe
	S2	50.41	Permissible	0.87	Safe
	S3	46.73	Permissible	0.92	Safe
	S4	37.91	Good	0.75	Safe
	S5	38.91	Good	0.75	Safe
	S6	37.37	Good	1.49	Marginally suitable
	S7	6.29	Excellent	1.47	Marginally suitable
	S8	5.21	Excellent	2.96	Not suitable
**Hand dug well**	**W1**	**51.35**	**Permissible**	**0.78**	**Safe**
	**W2**	**56.27**	**Permissible**	**0.65**	**Safe**
	**W3**	**71.53**	**Doubtful**	**0.35**	**Safe**
	**W4**	**68.16**	**Doubtful**	**1.39**	**Marginally suitable**
	**W5**	**17.18**	**Excellent**	**4.19**	**Not suitable**
Groundwater	B1	52.32	Permissible	0.97	Safe
	B2	71.25	Doubtful	0.80	Safe
	B3	60.73	Doubtful	0.75	Safe
	B4	47.66	Permissible	1.49	Marginally suitable
	B5	40.82	Permissible	1.49	Marginally suitable
	B6	1.40	Excellent	4.42	Not suitable
Max		71.25		4.42	
Min		1.40		0.74	
Mean		34.04	Excellent	1.54	Marginally suitable

#### Water hazard index

**Fig. 11:**
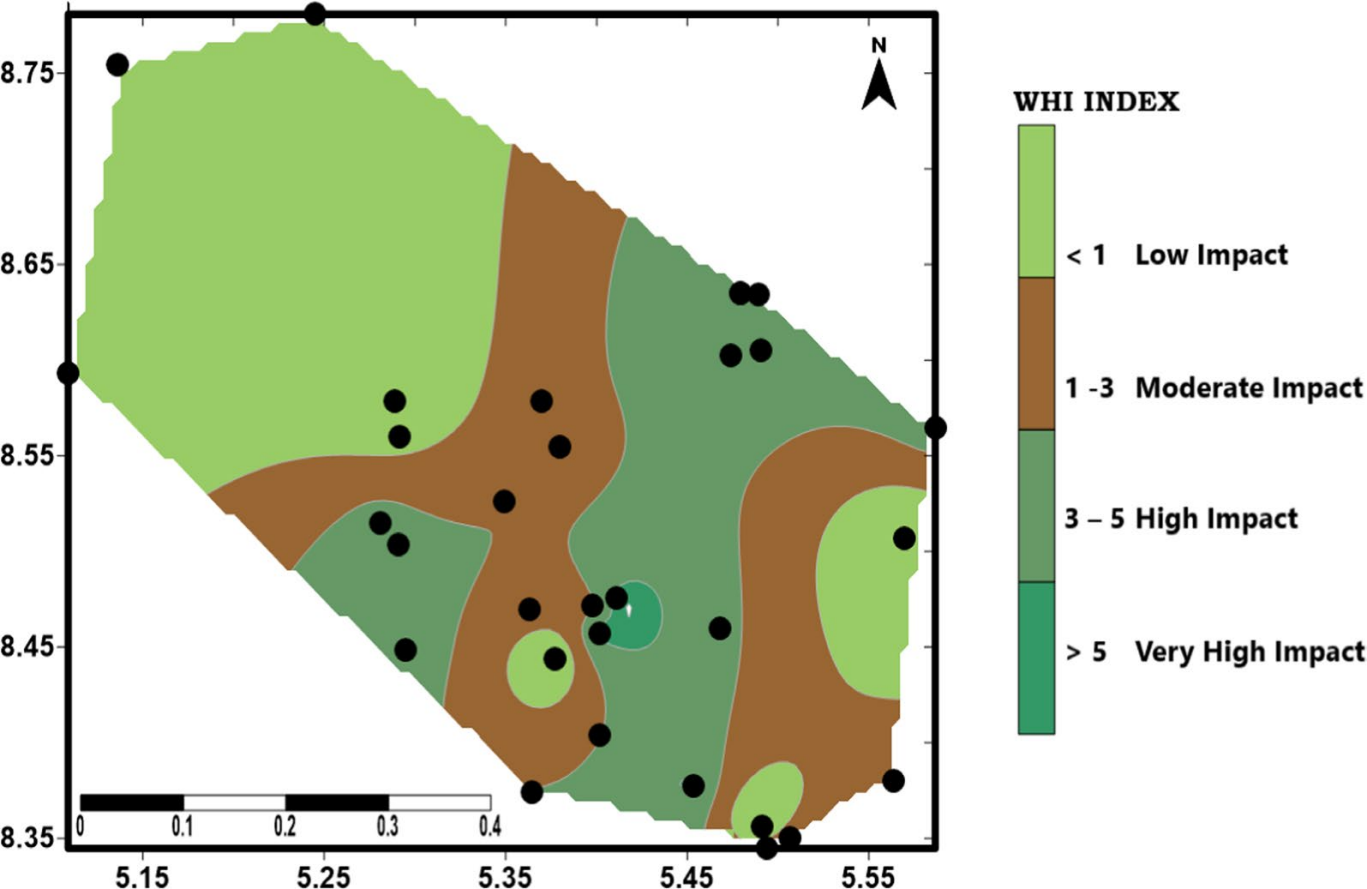
WHI map of the study area

The WHI has been used to provide an assessment of the overall quality of different types of water used for various purposes. The WHI was computed using parameters associated with quarrying activities that are known to negatively impact water quality. WHI were classified as follows: low impact (< 1), moderate impact (1–3), high impact (3–5) and very high impact (> 5) respectively. Generally, there is no defined pattern of distribution of the impact as the various water bodies are in the class of moderate to high impact. Figure 11 showed that 10% (S2, S4, S6) of the sites were classified as low impact, 36.67%( P4, P7, P8, P10, S1, S3, W3, W4, B2, B3, B4) of the sites were classified as moderate impact whereas 26.67%(P1,P2, P3, P6, P9, W1, B1, B5) of the sites were in high impact class and, 26.67%(P5, P11, S5,S7, S8, W3, W5, B6) of the sites are considered very high impact class respectively. The low impact areas are in the NW an SE part of the study area and are characterized by few quarry sites while the moderate to high-impact zone areas are sited in the central part of the study area where most of the quarries are located and are characterized by large amount of quarry wastes. Most of the sites were from the basement terrain with few locations (B6 and P11) in very high-impact zone in the sedimentary terrain. Geologically, both terrains have areas that are highly impacted implying that the quarry waste/rock fragment randomly scattered on the ground and dust generated during blasting and crushing and weathering of the rocks material are probable major source of the pollution as the metal(loid)s contained in the waste rocks and dust are leached from these materials into the water bodies and contaminate them.

### Health risk assessment

The fact that, the mean concentration of As, Cd, Pb, and Se are above the WHO [81] and SON [73] permissible limits for the different water resources is an indication that the population in the study area may be exposed to these potentially toxic metals. Long-term exposure to inorganic arsenic, especially through drinking water and food, can cause chronic arsenic poisoning. Many studies have shown the negative effects of arsenic on cognitive development, intelligence, and memory [75]. The International Agency for Research on Cancer (IARC) has classified arsenic and arsenic compounds as carcinogenic to humans. Arsenic is also associated with increased mortality in young adults due to many types of cancer, lung disease, disease, and kidney failure [31]. Exposure to elevated levels of concentration of Cd and Pb through drinking water pathways can lead to serious challenges for both humans and animals. Exposure to high concentrations of Cd, and Pb through drinking water can caused kidney disease, lung damage, high blood pressure, fragile bones and nervous disorder [6]. Also, exposure of infants and children to Pb concentrations above acceptable levels can delay physical and cognitive development and cause deficits in cognitive and learning abilities [6] (Environmental Protection Agency 2005). Health risk assessment of metal(loid)s in all the water samples from the study area were obtained by calculating the hazard quotients (HQ) and health risk index (HRI). HQ/HI < 1 is considered to have low health risk, HQ/HI > 1 are considered to have high health risk [58].

**Fig. 12:**
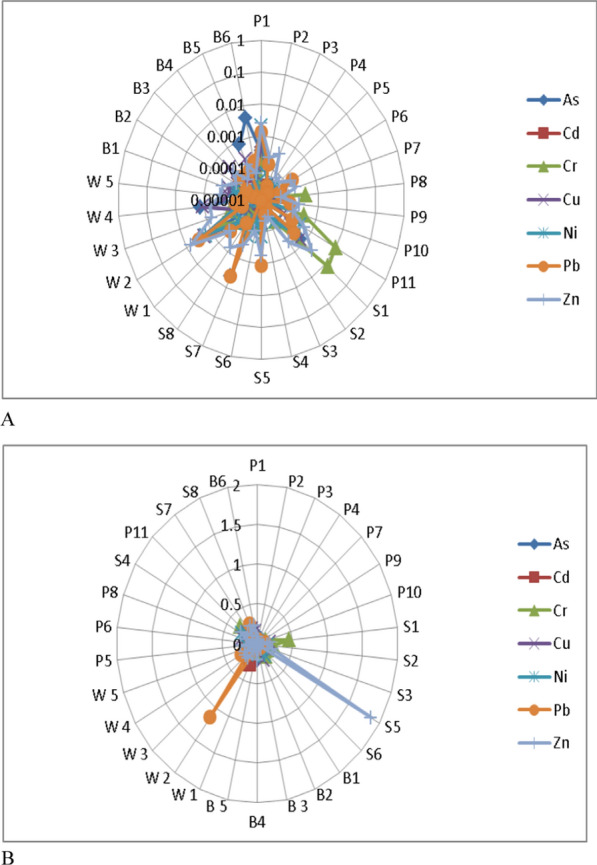
Radar plot showing spatial distribution of **A** ADD and **B** HQ values in the study area

**Fig. 13:**
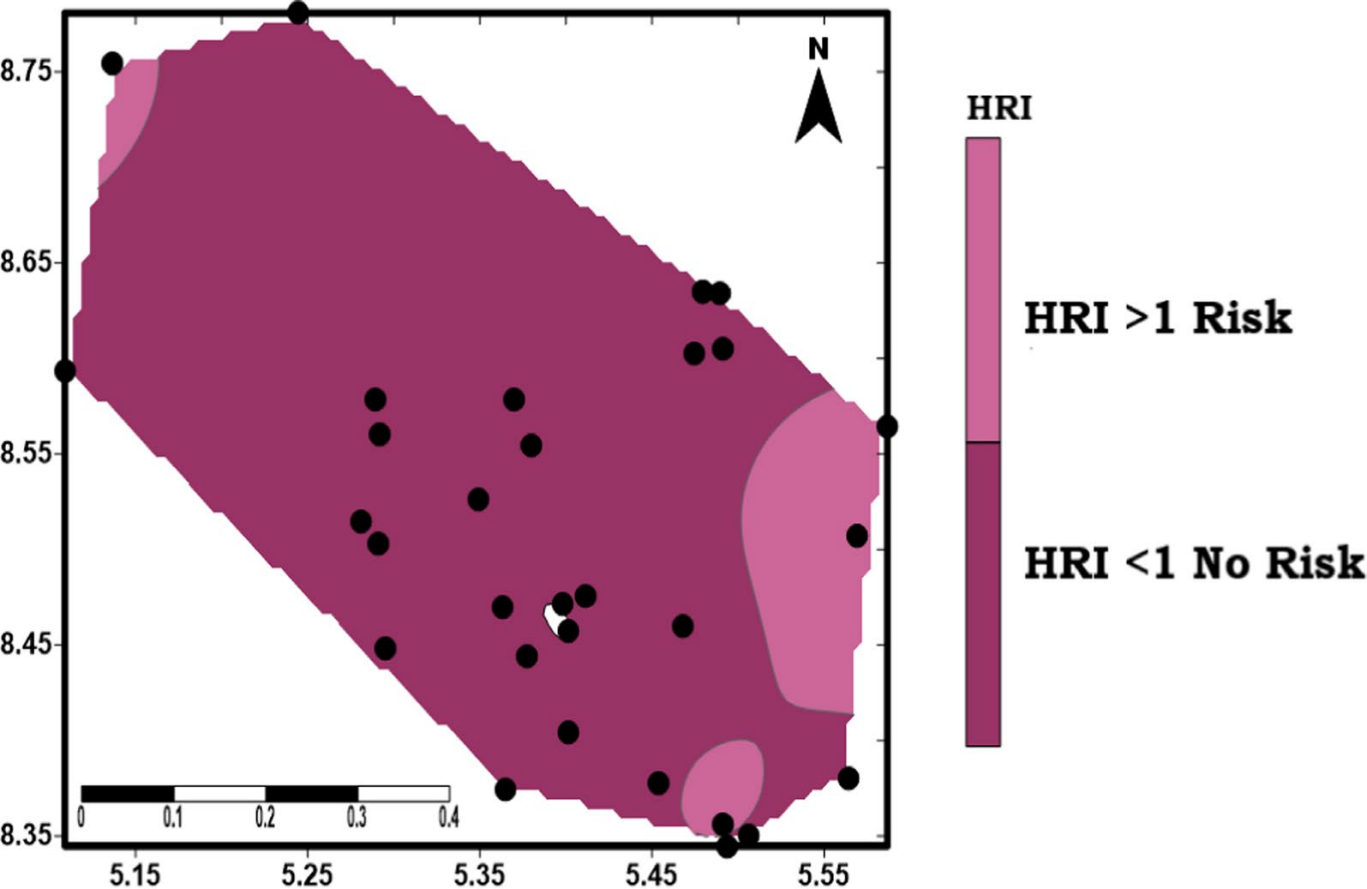
Health risk index(HRI) map

As, Cd, Cr, Cu, Ni, Pb, and Zn were considered in this study via drinking water pathways for the calculations of the ADD and HQ values (Figs. 12a and b). It was observed that the average daily dose (ADD) < 1 for all the locations (Fig. 12a) suggested low intake. However, HQ values for Zn (1.87) at S_5_ and Pb (1.14) via drinking water pathway at W2 is > 1 as well as the HRI (Fig. 13) of S_1_ (1.01), S_5_ (2.46) and W_2_ (1.67) all in the southeastern part of the study area are considered risky for ingestion. HQ and HRI values less than 1 indicate that there is no negative effect. However, areas with HQ value > 1 showed that the health of people living in these areas may be affected [38, 40, 80, 84].

Although HRI values were below 1 in some areas during the study period, long-term consumption of contaminated water from these sources may cause health problems in the future if water purification measures are not considered [38]. Lead exposure can cause diarrhea, anemia, gastrointestinal disturbances, sensitization, and progressive muscle paralysis [18]. Therefore, treatment is recommended.

## Conclusion

Investigation of the water resources near quarry sites in Akamkpa quarry district of southeastern Nigeria was carried out using hydrogeochemical, statistical, water hazard index (WHI), and health risk index. Many of the physicochemical parameters reflect differences in the composition of the water resources basically of natural, environmental, and anthropogenic origin. pH, DO values of P, S, W, and G were not within the acceptable limits of WHO [81] and SON [73]. The order of abundance of major anions in the water resources is HCO_3_—> Cl^−^ > SO_4_^2−^ > NO_3_^–^ and cations Ca^2+^  > Na^+^ > Mg^2+^  > K^+^.

The chemical composition due to major cations and anions showed that water resources were largely controlled by silicate weathering through ion exchange, dissolution, and some anthropogenic activities, mainly from nearby agricultural and quarrying activities. Principal component analysis and positive correlation with R > 0.7 between Ca^2+^ with HCO_3_^−^, Na^+^ with HCO_3_^−^ confirm their natural geogenic sources with silicate mineral dissolution as the main processes controlling the chemistry of the water resources. In addition, cross plots and saturation indices revealed that rock weathering including the dissolution of carbonates, and silicate minerals are significant rock-water interactions affecting the chemistry of the water resources.

However, except for some places, the water is classified as good and suitable for drinking and irrigation. Water samples in the study area were moderately and highly contaminated by As, Cd, Cr, Co, Mo, Pb, and Se probably from leaching of quarry waste materials and dust particles. Contamination of the pond water and boreholes is associated with As, Pb and Se while stream water is associated with Cr. The calculated water hazard index suggested that the water resources are mostly moderately to highly impacted. While a few places with HRI > 1 are considered risky. To reduce the health risks caused by drinking polluted water, local people should be informed by the operating companies as part of their social corporate responsibility on the importance of treating water to minimize health hazards associated with drinking polluted water.

Access to some of the quarry sites was not granted especially during operations like blasting to avoid injury, thus, it was not possible to collect quarry dust. There was no defined pattern of collection of the water samples and due to lack of funds, only 12 of the quarry sites were considered even though there were over thirty quarries.

Monitoring the water quality of the study area as well as proper location of the quarry sites is encouraged to reduce the risk of pollution. Additionally, environmentally friendly operation methods should be applied to properly manage the environment.

Hence further studies would be conducted by analyzing the quarry dust to establish the potential contribution of the quarry dust from quarrying operations to surface water alongside human activity.

## Supplementary Information

Supplementary material 1.

## Data Availability

Data generated has been used for this study and is available on demand from the corresponding author.
