# Supplementary material for: Investigation of hydrochemical characteristic, water quality and associated health risks of metals and metalloids in water resources in the vicinity of Akamkpa quarry district, southeastern, Nigeria
Source: Geochem Trans. 2024 Sep 12;25:7. doi: 10.1186/s12932-024-00090-y (PMC11395962; doi:10.1186/s12932-024-00090-y)
Supplement: Supplementary file 1 — Supplementary material 1. [file 12932_2024_90_MOESM1_ESM.doc]

**Supplementary material.**

**Investigation of hydrochemical characteristic, water quality and associated health risks of metals and metalloids in water resources in the vicinity of Akamkpa quarry district, southeastern, Nigeria.**

**Table 1: Hydro-chemical facies of the different water types in the study area**

| Geological |  |  |  |  |  |  |
| --- | --- | --- | --- | --- | --- | --- |
| Terrain | Water type | Sample code | Chemical facies | Water type | Sample code | Chemical facies |
| Oban Massif | Pond (P) | P_1_ | Ca-Mg-SO_4_-Cl | Pond (P) | P_5_ | Ca-Mg-SO_4_-Cl |
|  |  | P_2_ | Na-K-CO_3_-HCO_3_ |  | P_6_ | Ca-Mg-SO_4_-Cl |
|  |  | P_3_ | Ca-Mg-SO_4_-Cl |  | P_8_ | Ca-Mg-SO_4_-Cl |
|  |  | P_4_ | Na-K-HCO_3_ |  |  |  |
|  |  | P_7_ | Ca-Mg-SO_4_-Cl | Stream (S) | S_4_ | Na-K-HCO_3_ |
|  |  | P_9_ | Na-K-HCO_3_ |  |  |  |
|  |  | P_10_ | Ca-Mg-SO_4_-Cl |  |  |  |
|  | Stream (S) | S_1_ | Ca-Mg-SO_4_-Cl |  |  |  |
|  |  | S_2_ | Na-K-HCO_3_ |  |  |  |
|  |  | S_3_ | Na-K-HCO_3_ |  |  |  |
|  |  | S_5_ | Na-K-HCO_3_ |  |  |  |
|  |  | S_6_ | Na-K-HCO_3_ |  |  |  |
|  | Borehole (B) | B_1_ | Na-K-HCO_3_ |  |  |  |
|  |  | B _2_ | Na-K-HCO_3_ |  |  |  |
|  |  | B _3_ | Ca-Mg-SO_4_-Cl |  |  |  |
|  |  | B _4_ | Na-K-HCO_3_ |  |  |  |
|  |  | B _5_ | Na-K-HCO_3_ |  |  |  |
|  | Hand dug well (W) | W_1_ | Na-K-HCO_3_ |  |  |  |
|  |  | W _2_ | Ca-Mg-SO_4_-Cl |  |  |  |
|  |  | W _3_ | Na-K-HCO_3_ |  |  |  |
|  |  | W _4_ | Ca-Mg-SO_4_-Cl |  |  |  |
|  |  | W _5_ | Ca-Mg-SO_4_-Cl |  |  |  |
| Calabar Flank | Pond (P) | P_11_ | Ca-Mg-SO_4_-Cl |  |  |  |
|  | Stream (S) | S_7_ | Ca-Mg-SO_4_-Cl |  |  |  |
|  |  | S_8_ | Ca-Mg-SO_4_-Cl |  |  |  |
|  | Borehole (B) | B_6_ | Ca-Mg-SO_4_-Cl |  |  |  |

Appendix 2 a

A caculated contamination factor(Cf) of metal(loid)s of the water resources

Pond

Stream

Well

Borehole

Se

19.00

24.00

21.00

20.00

Fe

0.50

0.53

0.50

0.47

Mo

10.00

20.00

20.00

20.00

As

87.00

37.00

73.00

60.00

Co

2.00

1.00

1.00

2.00

Ni

0.05

0.50

0.50

0.50

Zn

0.02

0.01

0.01

0.01

Pb

42.00

12.00

15.00

44.00

Sb

0.50

0.40

0.30

0.30

Ag

0.70

0.30

0.70

0.50

Cd

80.00

10.00

0.00

76.67

Mn

0.00

0.05

0.00

0.05

Cu

1.00

0.02

0.01

0.02

Cr

6.80

7.20

8.40

0.20

< 1 low contamination, 1 - 10 medium contamination, > high contamination

|  |  |  |  |  |  |  |  |  |
| --- | --- | --- | --- | --- | --- | --- | --- | --- |
| 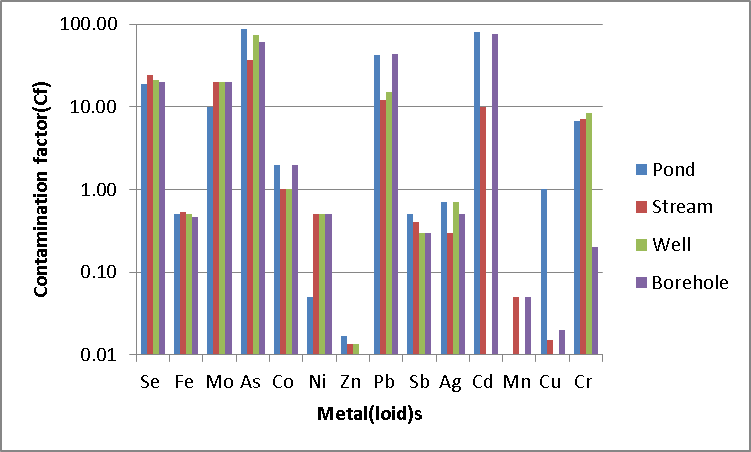   |  | | --- | |  |  |  |  |  |  |  |  |
|  |  |  |  |  |  |  |  |  |
|  |  |  |  |  |  |  |  |  |
|  |  |  |  |  |  |  |  |  |
|  |  |  |  |  |  |  |  |  |
|  |  |  |  |  |  |  |  |  |
|  |  |  |  |  |  |  |  |  |
|  |  |  |  |  |  |  |  |  |
|  |  |  |  |  |  |  |  |  |
|  |  |  |  |  |  |  |  |  |
|  |  |  |  |  |  |  |  |  |
|  |  |  |  |  |  |  |  |  |
|  |  |  |  |  |  |  |  |  |
|  |  |  |  |  |  |  |  |  |
|  |  |  |  |  |  |  |  |  |
|  | <1 low contamination, 1- 10 medium contamination, > 10 high contamination | | | | | | | |
|  | Appendix 2b plot of contamination factor against metal(loid)s of water resources in the study area. | | | | | | |  |
|  |  |  |  |  |  |  |  |  |
